# A small molecule inhibitor of the UBE2F-CRL5 axis induces apoptosis and radiosensitization in lung cancer

**DOI:** 10.1038/s41392-022-01182-w

**Published:** 2022-10-17

**Authors:** Tiantian Xu, Qisheng Ma, Yanan Li, Qing Yu, Peichen Pan, Yawen Zheng, Zhijian Li, Xiufang Xiong, Tingjun Hou, Bin Yu, Hongmin Liu, Yi Sun

**Affiliations:** 1 Cancer Institute, the Second Affiliated Hospital, and Institute of Translational Medicine, Zhejiang University School of Medicine, Hangzhou, 310029 China; 2 Cancer Center, Zhejiang University, Hangzhou, 310058 China; 3 Research Center for Life Science and Human Health, Binjiang Institute of Zhejiang University, Hangzhou, 310053 China; 4 School of Pharmaceutical Sciences, Key Laboratory of Advanced Drug Preparation Technologies, Military of Education, Zhengzhou University, Zhengzhou, 450001 China; 5 College of Pharmaceutical Sciences, Zhejiang University, Hangzhou, 310058 China; 6 Department of Oncology, Jinan Central Hospital, Cheeloo College of Medicine, Shandong University, Jinan, 250033 China

**Keywords:** Drug development, Lung cancer, Drug discovery

## Abstract

Protein neddylation is catalyzed by a neddylation activating enzyme (NAE, E1), an E2 conjugating enzyme, and an E3 ligase. In various types of human cancers, the neddylation pathway is abnormally activated. Our previous study validated that the neddylation E2 UBE2F is a promising therapeutic target in lung cancer. Although the NAE inhibitor MLN4924/pevonedistat is currently under clinical investigation as an anti-cancer agent, there are no small molecules available that selectively target UBE2F. Here, we report, for the first time, the discovery, via structure-based virtual screen and chemical optimization, of such a small molecule, designated as HA-9104. HA-9104 binds to UBE2F, reduces its protein levels, and consequently inhibits cullin-5 neddylation. Blockage of cullin-5 neddylation inactivates cullin-RING ligase-5 (CRL5) activity, leading to accumulation of the CRL5 substrate, NOXA, to induce apoptosis. Moreover, HA-9104 appears to form the DNA adduct via its 7-azaindole group to induce DNA damage and G2/M arrest. Biologically, HA-9104 effectively suppresses the growth and survival of lung cancer cells and confers radiosensitization in both in vitro cell culture and in vivo xenograft tumor models. In summary, we discovered a small molecule, designated HA-9104, that targets the UBE2F-CRL5 axis with anti-cancer activity alone or in combination with radiation.

## Introduction

Protein modification via neddylation regulates the stability, activity, or function of substrate proteins by covalently attaching a ubiquitin-like peptide NEDD8 (neural precursor cell expressed developmentally downregulated protein 8) to a substrate protein. The physiological substrates of neddylation modification are cullins, a family of proteins, serving as the molecular scaffolds responsible for assembling cullin-RING ligases (CRLs).^1,2^ Typical neddylation modification is catalyzed sequentially by three enzymes. The NEDD8 is activated by an E1 NEDD8-activating enzyme in the presence of ATP, then transferred to an E2 NEDD8-conguating enzyme via a thioester bond. Finally, an E3 ligase binds both NEDD8-loaded E2 and a substrate (e.g. cullin) to promote the covalent attachment of NEDD8 to the lysine residue on the cullin, leading to activation of CRLs.^3,4^ In mammalian cells, there is a single E1 (NAE), consisting of a catalytic subunit, UBA3/NAEβ and a regulatory subunit, APPBP1/NAE1; two E2s UBE2F and UBE2M (also known as UBC12), and several E3s. UBE2F couples with SAG/RBX2 to promote neddylation of cullin-5, whereas UBE2M couples with RBX1 to promote neddylation of cullins 1–4.^5^ Cullin neddylation triggers a conformational change of cullins to activate CRLs. CRLs are multi-component-containing and the largest family of E3 ubiquitin ligases, which degrade ~20% of cellular proteins doomed for proteasome degradation through UPS, thus regulating many key biological processes, including cell cycle progression, DNA replication and repair, signal transduction, and tumorigenesis.^6,7^

A wealth of data from many studies have accumulated in the past decade, showing that in many human cancers, the neddylation pathway is over-activated via overexpression of NEDD8, UBE2F, UBE2M, SAG/RBX2, which is often associated with poor patient survival.^8–13^ Thus, the neddylation pathway can serve as an attractive anti-cancer target.^12–15^ Indeed, MLN4924, also known as Pevonedistat, is a first-in-class NAE inhibitor^16^ that has been in many phase I-III clinical trials for patients with leukemia, lymphoma, melanoma, and several advanced solid tumors, including a phase II trial of pevonedistat plus docetaxel in patients with advanced non-small cell lung cancer (NCT03228186), based upon our previous study.^10^ Given the fact that MLN4924, as a NAE inhibitor, inhibits the entire neddylation pathway, which is essential for many physiological processes, its cytotoxic side-effect appears unavoidable. Up to now, 13 years since the first report,^16^ pevonedistat is still an investigational drug of which the efficacy and safety have not been fully demonstrated. In July 2020, FDA granted pevonedistat as breakthrough therapy designation for the treatment of patients with higher-risk myelodysplastic syndromes (HR-MDS) (https://www.takeda.com/newsroom/newsreleases/2020/takeda-announces-u.s.-fda-breakthrough-therapy-designation-granted-for-pevonedistat-for-the-treatment-of-patients-with-higher-risk-myelodysplastic-syndromes-hr-mds/). However, the Phase III PANTHER (Pevonedistat-3001) study was recently ended for not achieving the primary endpoint of event-free survival at the statistically significant level (https://www.takeda.com/newsroom/newsreleases/2021/takeda-provides-update-on-phase-3-panther-pevonedistat-3001-trial/). Therefore, the discovery of small molecule inhibitors that target down-stream enzyme in neddylation pathway should, in theory, have improved specificity and selectivity with reduced side-effects.

Our recent study validated neddylation E2 UBE2F as an attractive target for lung cancer.^11^ However, no small molecule inhibitor of UBE2F, to the best of our knowledge, has been reported. In this study, we report the discovery of such a small molecule, designated HA-9104, via a structure-based virtual screen and multiple rounds of chemistry-based optimization. HA-9104 has potent growth suppression and radiosensitizing activities via targeting the UBE2F-CRL5 axis and causing DNA damage, leading to induction of apoptosis and G2/M arrest in lung and pancreatic cancer cells. Thus, HA-9104 may serve as a chemical prototype for future development into a new class of anti-cancer agents or radiosensitizers.

## Results

### Discovery of HA-9104 as a novel small molecule inhibitor targeting UBE2F

**Fig. 1:**
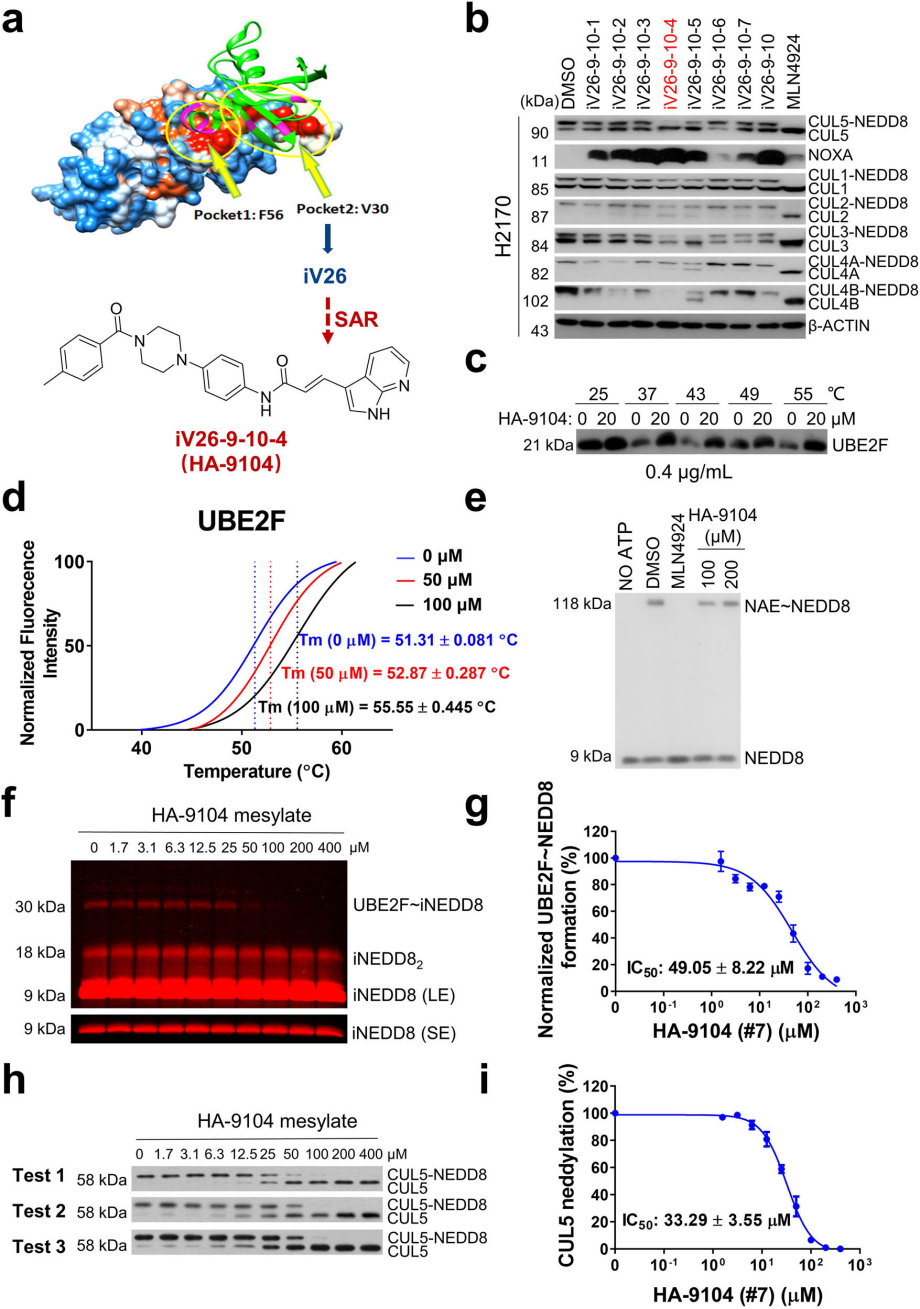
Discovery of HA-9104, which binds to UBE2F, alters its thermal stability and inhibits cullin-5 neddylation in vitro. **a** The flow chart for the discovery of HA-9104 aims at targeting the V30 pocket of UBE2F. **b** Selective inhibition of cullin-5 neddylation by **iV26-9-10-4** (HA-9104). H2170 cells were treated with the derivatives of **iV26-9-10** (20 μM) for 24 h and then harvested for Western blotting. **c** Enhancement of thermo-stability of UBE2F by HA-9104. Purified UBE2F (0.4 μg/mL) was incubated with HA-9104 (20 μM) at 25 °C for 10 min in 50 μL buffer and then heated at the indicated temperature for 5 min. UBE2F protein levels were measured by Western blotting. **d** Enhancement of Tm of UBE2F by HA-9104. The assay was performed as described in M&M. The S-shaped thermal denaturation curves were generated and applied to Boltzmann Equation to identify the Tm at the midpoint of the unfolding transition. **e** The in vitro inhibition of NAE~NEDD8 thioester. HA-9104-mesylate at indicated concentrations or MLN4924 was incubated with a reaction mixture, containing purified E1 (UBA3/APPBP1) and NEDD8, followed by Western blotting. **f**, **g** The in vitro inhibition of UBE2F~NEDD8 thioester. HA-9104-mesylate at indicated concentrations was incubated with a reaction mixture, containing purified E1 (UBA3/APPBP1), E2 (UBE2F), and iNEDD8 (iFluor 680-labeled NEDD8), followed by SDS-PAGE gel. The gel was scanned and quantitated by the ODYSSEY Infrared Imaging System, *n* = 3. SE shorter exposure, LE longer exposure. **h**, **i** The in-vitro inhibition of cullin-5 neddylation. HA-9104-mesylate at indicated concentrations was incubated with a neddylation reaction mixture, containing purified E1 (UBA3/APPBP1), E2 (UBE2F), E3/substrate (SAG/CUL5), and NEDD8, followed by Western blotting. The band density was quantified by Image J software, *n* = 3. Shown is mean ± SD

In an effort to identify small molecules with the potential to disrupt the UBE2F-UBA3 interaction via targeting two binding pockets (F56 and V30) on the UBE2F surface,^17^ we conducted a structure-based virtual screen of Specs chemical library (http://www.specs.net/) with a total of 240,000 compounds, and have identified HA-1141, a small molecule NAE E1 inhibitor targeting the F56 pocket.^17^ Here, we report the discovery, structural optimization, and characterization of HA-9104, a novel small molecule inhibitor of cullin-5 neddylation via virtually targeting the V30 pocket of UBE2F (Fig. 1a). A total of 90 top-ranked compounds identified from the virtual screen and 72 of their homologs were screened via the Western blotting for their potential inhibition of cullin-5 neddylation, a consequence of UBE2F inhibition^5^ in H358 lung cancer cells after a 24-h treatment at 20 μM. Among them, the compound **iV26** showed the best activity (shown in Supplementary Fig. 1a is 12 such compounds with MLN4924 as positive control) and thus was chosen as a hit compound for further optimizations. For the first round of structural modifications, we took two approaches: First, we modified the 3-chlorobenzoyl group with diverse acyl groups (the R^1^ group), while retaining the 4-methylbenzoyl group intact, leading to 14 derivatives (**iV26-1** to **9**, **16**, **18**, **19**, **22**, **25**). Second, we modified the 4-methylbenzoyl group attached to the piperazine ring with three different acyl groups to generate 11 derivatives (**iV26-10** to **15**, **17**, **20**, **21**, **23**, **24**) (Supplementary Fig. 1b). The follow-up Western blotting revealed that compound **iV26-9** bearing the cinnamon acyl moiety (Supplementary Fig. 1c) was the best one on both inhibiting cullin-5 neddylation and causing accumulation of NOXA, a known substrate of CRL5^11^ (Supplementary Fig. 1d). The further structural optimizations were focused on the introducing different cinnamon acyl moieties, leading to the discovery of **iV26-9-10** bearing an indole ring (Supplementary Fig. 1e), which showed the best activity in inducing NOXA accumulation in both H358 and H2170 lung cancer cells with partial inactivation of cullin-5 neddylation (Supplementary Fig. 1f). The final optimization was focused on the indole ring with different substitutions on the indole ring or nitrogen-hybridized indole ring, and identified **iV26-9-10-4** (Fig. 1a) as the best derivative in both selectively inhibiting neddylation of cullin-5 among other cullin family members, and causing remarkable NOXA accumulation (Fig. 1b). The **iV26-9-10-4** was designated as HA-9104 and was used in the rest of the studies.

To determine the potential binding between HA-9104 and UBE2F in vitro, we used a mesylate form of HA-9104 (Supplementary Fig. 5b) to increase compound solubility. By an in-vitro thermal stability assay, we found that purified UBE2F is rather heat-liable and HA-9104 stabilizes it in all temperatures tested (Fig. 1c). We next performed an in vitro fluorescent thermal shift assay (also called differential scanning fluorimetry, DSF),^18,19^ with continuous temperature increases, and found that HA-9104 caused an increase in melting temperature of UBE2F up to 4 °C in a dose-dependent manner (Fig. 1d).

To determine the exact step of NEDD8 transfer that is inhibited by HA-9104, we set up three in-vitro neddylation assays, using purified proteins in the test tubes, to measure the formation of E1~N8 thioester, E2~N8 thioester, and cullin-5 neddylation, mimicking each step of the enzymatic cascade of substrate neddylation. We first validated our assay using MLN4924, a well-characterized inhibitor of neddylation E1,^16^ as a positive control. Expectedly, MLN4924 completely inhibited the formation of E1~N8 thioester, but HA-9104 had no such effect at the concentration up to 200 μM (Fig. 1e), indicating that HA-9104 did not target neddylation E1. We next established an E2~N8 thioester assay using purified E1, E2, and NEDD8 in a reaction mixture containing DMSO or increasing concentrations of HA-9104, and found that HA-9104 indeed caused a dose-dependent inhibition of E2/UBE2F~N8 thioester (Fig. 1f) with an IC_50_ at ~49 μM (Fig. 1g). Finally, we measured in vitro cullin-5 neddylation in a reaction mixture, containing purified neddylation E1, E2, E3, NEDD8, and various concentrations of HA-9104 and found that HA-9104 inhibited cullin-5 neddylation in a dose-dependent manner (Fig. 1h) with an IC_50_ of ~30 μM (Fig. 1i). Taken together, these in vitro assays clearly demonstrate that HA-9104 inhibits cullin-5 neddylation via blocking the formation of UBE2F~NEDD8 thioester.

To determine possible UBE2F pocket(s) onto which HA-9104 binds, we used *Glide* docking simulations to predict the binding geometries^20^ and identified three top binding pockets, ranking from the highest to the lowest: N152 (docking score of −4.7), V98 (docking score of −3.862) and V30 (docking score of −3.325). The N152 pocket involved HA-9104 binding residues of Leu151, Asp154, Phe153, Asn152, His165, Ala162, and Ile159, with residues Asp154 and His165 forming two hydrogen bonds, and Phe153 forming cation-π interaction with HA-9104 (Supplementary Fig. 1g, left panel). The V98 pocket involved residues Arg121, Leu120, Lys97, Val98, Thr112, Cys116 (catalytic cysteine residue), Ile115, Glu114, and Lys99 for HA-9104 binding with three hydrogen bonds (two side chains of Lys97 and Lys99, and one backbone on Ile115), and one cation-π interaction on Arg121 (Supplementary Fig. 1g, middle panel). The V30 pocket involved residues Asn60, Lys61, His63, Asp34, Lys35, and Lys39 with the formation of two hydrogen bonds involving Asp34 side chain and Asn60 backbone (Supplementary Fig. 1g, right panel).

We then made three UBE2F mutants targeting these sites, respectively, by replacing the surrounding amino acid residues on these sites with residue alanine to disrupt the hydrogen bonds and cation-π interaction between HA-9104 and UBE2F. These mutants were N152 (F153A/D154A/H165A), V98 (K97A/K99A/R121A), and V30 (D34A). Note that it is predicted to be impossible to disrupt the hydrogen bond between HA-9104 and backbone residues Ile115 (V98 pocket) and Asn60 (V30 pocket). These residues were, therefore, excluded from these mutants.

We then determined the thermo-stability of these mutants in comparison with wt (wild-type) UBE2F at a very high final concentration of 40 µg/mL, using Coomassie brilliant blue staining, and found that the V30 mutant had a significantly reduced thermo-stability (Supplementary Fig. 1h), which was excluded for further analysis to keep a fair comparison with UBE2F-wt. We next performed the in vitro thermal shift assay using Western blotting on UBE2F-wt and UBE2F-V98 and UBE2F-N152 mutants in the absence and presence of HA-9104. The results showed that compared to UBE2F-wt, N152 mutant was very thermo-stable and largely resistant to HA-9104-induced stabilization, whereas V98 mutant was a little more thermo-stable and less stabilized by HA-9104 (Supplementary Fig. 1i). We further compared the enzymatic activity between UBE2F-wt and two mutants in catalyzing cullin-5 neddylation, and found that both mutants, particularly V98 (near catalytic cysteine residue/Cys116), had significantly reduced catalytic activity (Supplementary Fig. 1j, k), an effect similar to HA-9104 inhibition, suggesting V98 and N152 pockets play the critical role to facilitate cullin-5 neddylation. Taken together, these results suggest that HA-9104 likely binds to UBE2F through the N152 and/or V98 pockets.

### HA-9104 reduces UBE2F protein levels to selectively inhibits cullin-5 neddylation

**Fig. 2:**
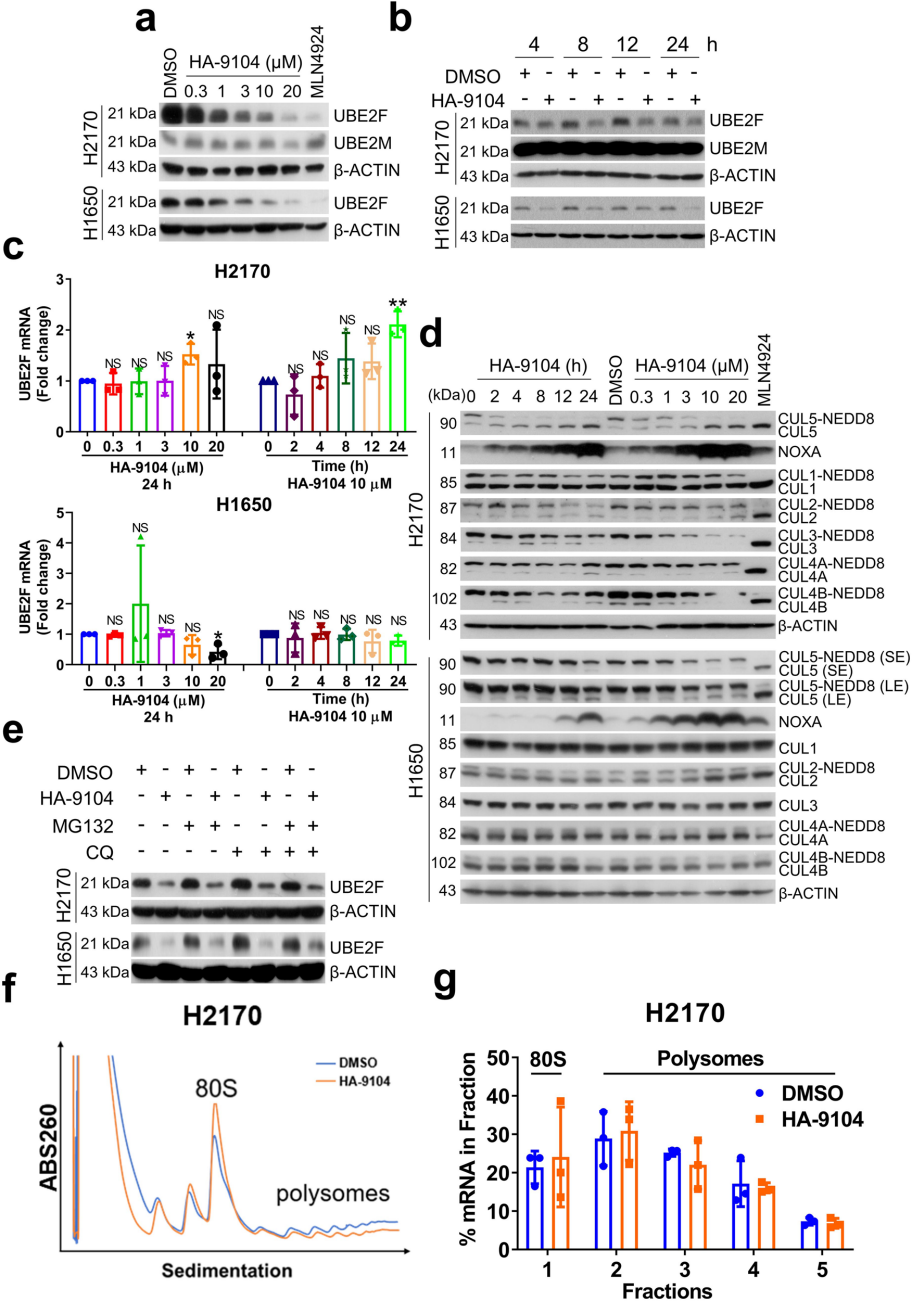
HA-9104 reduces UBE2F protein levels. **a**, **b** HA-9104 reduces the levels of UBE2F protein. H2170 and H1650 cells were treated with HA-9104 at various concentrations for 24 h, or MLN4924 (0.3 μM) as a positive control (**a**), or treated with DMSO or HA-9104 (10 μM) for indicated time periods (**b**), and then harvested for Western blotting. **c** HA-9104 treatment has no or a weak effect on UBE2F mRNA level. H2170 and H1650 cells were treated with HA-9104 at various concentrations for 24 h (left), or treated with HA-9104 (10 μM) for indicated time periods (right), followed by qRT-PCR, *n* = 3. **d** HA-9104 preferentially inhibits cullin-5 neddylation and induces NOXA accumulation. H2170 and H1650 cells were treated with HA-9104 (20 μM) for indicated time periods (left) or indicated concentrations of HA-9104 for 24 h (right), and then harvested for Western blotting. SE shorter exposure, LE longer exposure. **e** HA-9104 does not affect UBE2F turnover. H2170 and H1650 cells were treated with HA-9104 (10 μM), along with DMSO control for 24 h. MG132 (10 μM) or/and CQ (50 μM) were added 6 h before harvest, followed by Western blotting. **f**, **g** HA-9104 inhibits global translation, but not UBE2F translation. H2170 cells were treated with HA-9104 (10 μM) or DMSO control for 24 h, then subjected to ribosome profiling. **f** A representative curve of mRNA translation. **g** The qRT-PCR result of UBE2F mRNA, *n* = 3. Shown is mean ± SD. NS not significant, *p < 0.05, **p < 0.01

Next, we determined the effect of HA-9104 on the cellular levels of UBE2F in several lines of lung cancer cells. HA-9104 was found to reduce the protein levels of UBE2F in a dose-dependent (Fig. 2a, Supplementary Fig. 2a), as well as a time-dependent manner (Fig. 2b, Supplementary Fig. 2b), while having no or moderate effect on UBE2F mRNA (Fig. 2c, Supplementary Fig. 2c). Interestingly, HA-9104 did not affect the protein levels of UBE2M, a family member of UBE2F (Fig. 2a, b, Supplementary Fig. 2a, b, note that UBE2M was not detectable in H1650 cells), suggesting its selectivity toward UBE2F. Given UBE2F couples with RBX2/SAG to promote cullin-5 neddylation, whereas UBE2M couples with RBX1 to promote neddylation of cullins 1-4,^5^ the selectivity of HA-9104 toward UBE2F would suggest its selectivity to inhibit cullin-5 neddylation. Indeed, in all three lung cancer lines and one pancreatic cancer line tested, HA-9104 preferentially inhibited cullin-5 neddylation and caused accumulation of CRL5 substrate NOXA, in both dose- and time-dependent manners, with minor, if any, inhibition of cullins 1-4, the other cullin family members (Fig. 2d, Supplementary Fig. 2d, e).

We then investigated the possible mechanism by which HA-9104 reduced the levels of UBE2F protein. We first determined whether HA-9104 enhanced UBE2F protein degradation, and found that it was not the case, since either proteasome inhibitor MG132 or lysosome inhibitor CQ (chloroquine) or even in combination failed to rescue UBE2F reduction by HA-9104 (Fig. 2e, Supplementary Fig. 2f). Given HA-9104 did not affect UBE2F mRNA, nor UBE2F degradation, we next determined whether HA-9104 affected the translation of UBE2F protein, using the ribosome profiling analysis. In comparison to DMSO control, HA-9104 treatment caused a minor-to-moderate increase of the 80S monosome peak, but a moderate decrease of the polysome peaks (Fig. 2f, Supplementary Fig. 2g), suggesting that HA-9104 may have a general impairment effect on mRNA translation. However, UBE2F translation appeared not to be inhibited, since the qRT-PCR analysis of each fraction showed an equal amount of UBE2F mRNA regardless of HA-9104 treatment (Fig. 2g, Supplementary Fig. 2h). Taken together, it appears that HA-9104 reduces UBE2F protein via a mechanism of neither enhanced degradation, nor reduced translation.

The ribosomal profile experiments suggested that HA-9104 may have a general impairment effect on mRNA translation. We next carried out a quantitative proteomic analysis to evaluate the inhibition selectivity of HA-9104. H2170 cells were treated with HA-9104 at 10 µM for 24 h, followed by proteomic profiling analysis. Only 46 proteins were identified with a twofold reduction (Supplementary Fig. 2i) (for more details, see http://www.proteomexchange.org with the dataset identifier no. PXD036191). Thus, it appears that HA-9104 is not a general inhibitor of protein synthesis.

### HA-9104 significantly induced apoptosis via NOXA accumulation

**Fig. 3:**
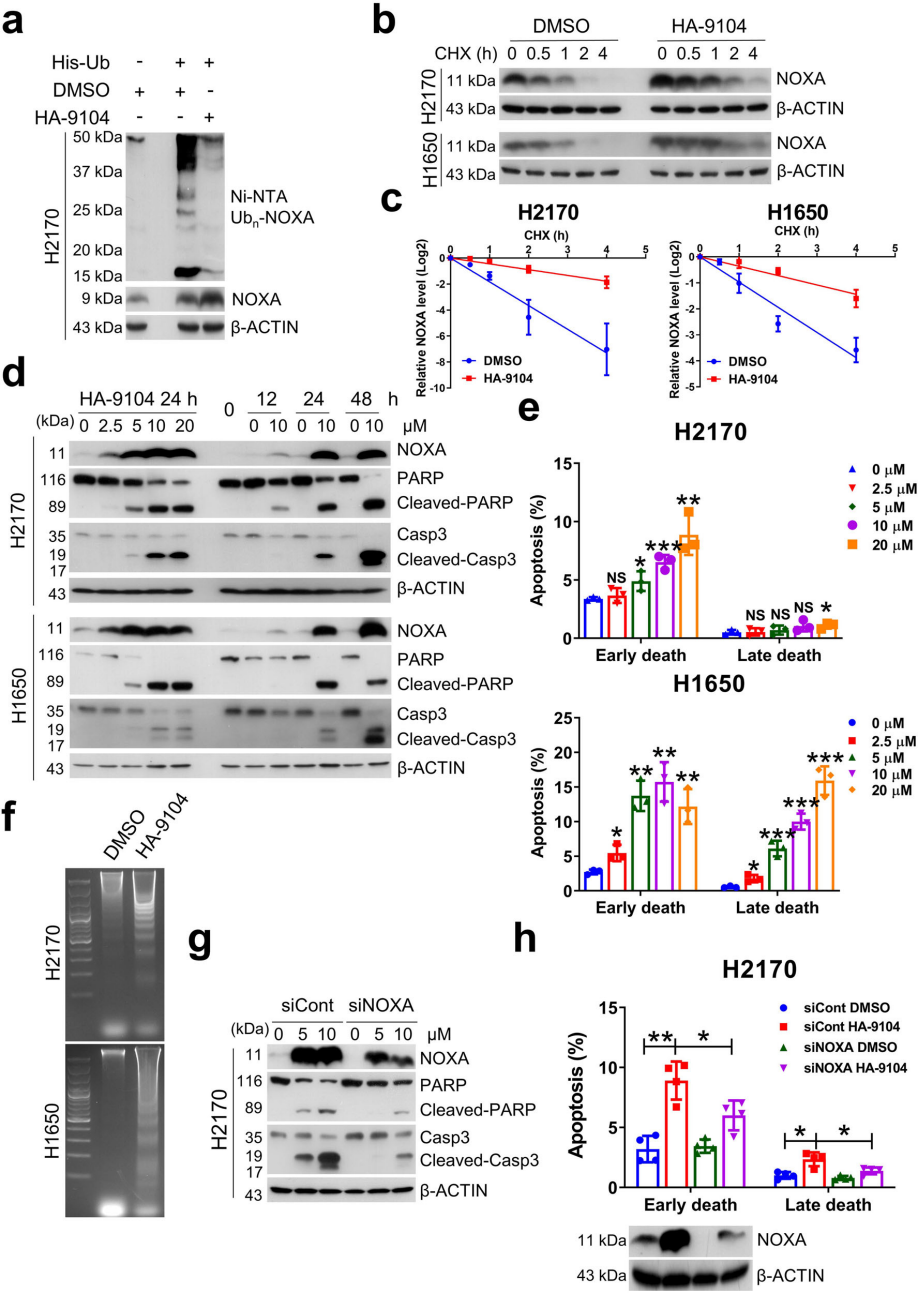
HA-9104 causes NOXA accumulation to induce apoptosis. **a** HA-9104 inhibits NOXA polyubiquitination. H2170 cells were transfected with His-tagged ubiquitin for 24 h, then treated with HA-9104 (10 μM), along with DMSO control for another 24 h. Cell lysates were subjected to in vivo ubiquitylation assay as described in M&M. **b, c** HA-9104 prolongs NOXA protein half-life. H2170 and H1650 cells were treated with HA-9104 (10 μM) or DMSO control in the presence of CHX (50 μg/mL) for indicated time periods, followed by Western blotting. The band density was quantified by Image J software, *n* = 3. **d**–**f** HA-9104 induces apoptosis in a dose- and time-dependent manner. H2170 and H1650 cells were treated with various concentrations of HA-9104 for 24 h (left), or with HA-9104 (10 μM) for indicated time periods (right), and then subjected to Western blotting (**d**), combined annexin V-FITC and PI staining (**e**), and DNA fragmentation assay (**f**). **g**, **h** Silencing of NOXA partially rescues apoptosis induced by HA-9104. H2170 cells were transfected with siCont or siNOXA for 48 h and then treated with HA-9104 (5, 10 μM) or DMSO control for another 24 h, followed by Western blotting (**g**) or combined annexin V-FITC and PI staining, *n* = 4 (**h**). Shown is mean ± SD. NS not significant, *p < 0.05, **p < 0.01, ***p < 0.001

We next determined whether HA-9104-induced NOXA accumulation was due to reduced degradation as a result of inhibition of cullin-5 neddylation. Indeed, HA-9104 treatment significantly inhibited NOXA polyubiquitination (Fig. 3a, Supplementary Fig. 3a) and prolonged NOXA half-life (Fig. 3b, c, Supplementary Fig. 3b, c) with no or moderate effect on NOXA mRNA in all three lung cancer cell lines tested (Supplementary Fig. 3d).

Since NOXA is a pro-apoptotic protein, we determined the biological consequence of NOXA accumulation. In all three lung cancer cell lines tested, HA-9104 induced significant apoptosis in the dose- and time-dependent manners, as demonstrated by cleavage of PARP and caspase-3 (Fig. 3d, Supplementary Fig. 3e), Annexin V-FITC/PI FACS analysis (Fig. 3e, Supplementary Fig. 3f), and DNA fragmentation (Fig. 3f, Supplementary Fig. 3g). HA-9104 also induced apoptosis in MIAPaCa-2 cells, as evidence by obvious PARP cleavage (Supplementary Fig. 2e). Finally, we investigated if accumulated NOXA was causally associated with apoptosis induction via siNOXA-based rescue experiments and found that NOXA knockdown indeed significantly attenuated the degree of apoptosis (Fig. 3g, h, Supplementary Fig. 3h, i), indicating HA-9104-induced apoptosis is mainly mediated by accumulated NOXA.

### HA-9104 induces G2/M arrest and triggers replication stress to delay DNA damage repair

**Fig. 4:**
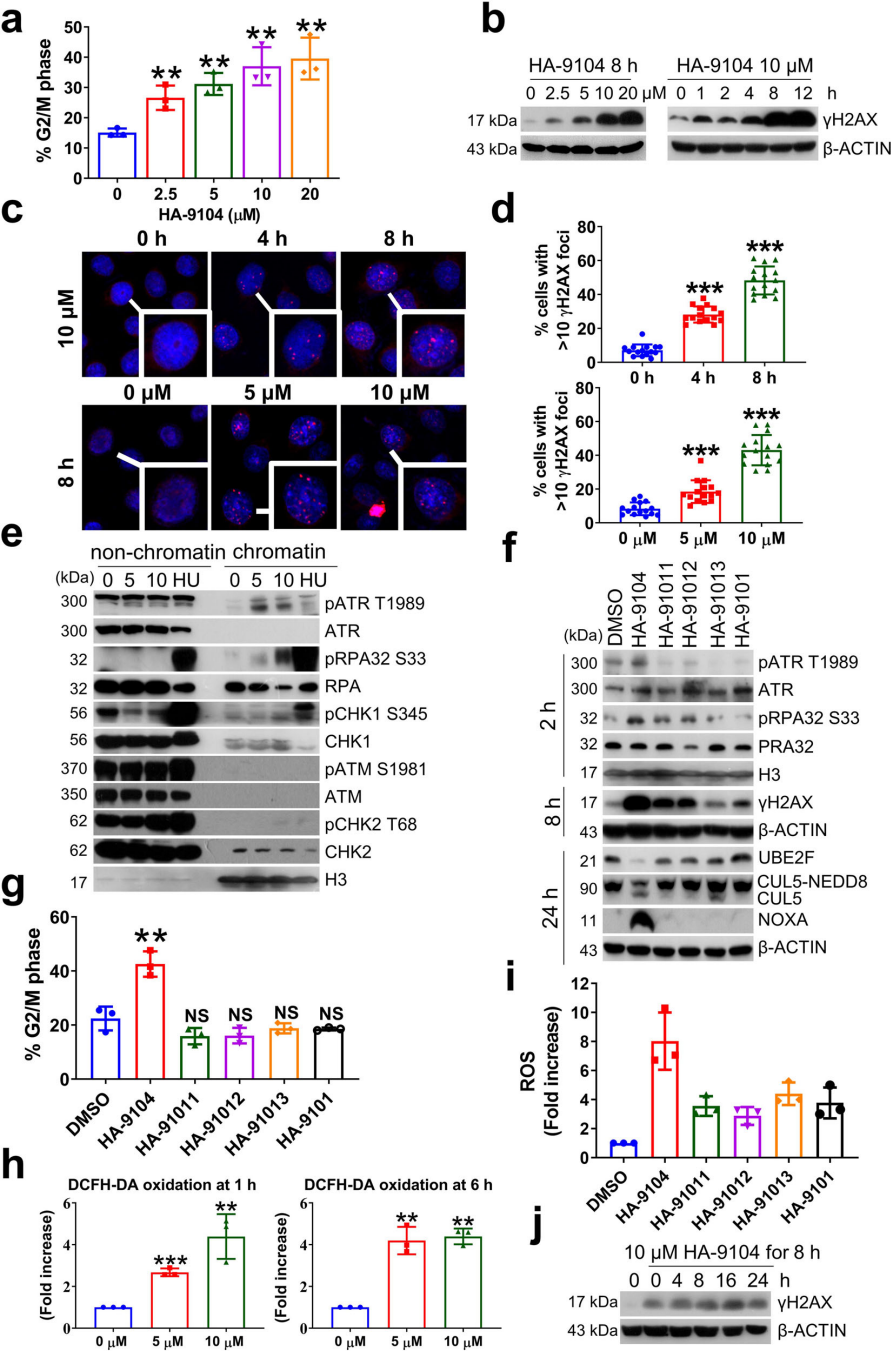
HA-9104 induces DNA damage to trigger G2/M arrest. **a** HA-9104 causes G2/M arrest. H1650 cells were treated with various concentrations of HA-9104 for 24 h, followed by FACS analysis. The histogram of cell percentage in G2/M phase was shown. *n* = 3. **b** HA-9104 causes γH2AX accumulation. H1650 cells were treated with HA-9104 (10 μM) for indicated time periods (left) or indicated concentrations of HA-9104 for 8 h (right), and then harvested for Western blotting. **c**, **d** HA-9104 causes γH2AX foci in the nucleus. H1650 cells were treated with HA-9104 (10 μM) for indicated time periods (up) or indicated concentrations of HA-9104 for 8 h (down). γH2AX foci were observed by immunofluorescence. **e** HA-9104 treatment activates the ATR pathway. H1650 cells were treated with HA-9104 (5, 10 μM) for 2 h. Hydroxyurea (HU) (5 mM) was used as a positive control. Chromatin proteins were isolated from whole cell lysate, followed by Western blotting. **f**, **g** Comparison of HA-9104 with its derivatives. H1650 cells were treated with indicated drugs (10 μM) for indicated time periods, then analyzed for the activation of the ATR pathway (2 h), γH2AX induction (8 h), UBE2F reduction, and cullin-5 neddylation inhibition (24 h) (**f**), and G2/M arrest (24 h), *n* = 3 (**g**). **h**, **i** HA-9104, rather than its derivatives, induces ROS production in a dose-dependent manner. H1650 cells were treated with HA-9104 (5, 10 μM) for indicated time periods (**h**), or treated with indicated drugs (10 μM) for 6 h (**i**). ROS was labeled using DCFH-DA and then analyzed through FACS. *n* = 3. **j** HA-9104 induces irreversible DNA damage. H1650 cells were treated with HA-9104 (10 μM) for 8 h first, and then washed with PBS 3 times and replaced with fresh medium without HA-9104 for indicated time periods. γH2AX levels were determined by Western blotting. Shown is mean ± SD. NS not significant, *p < 0.05, **p < 0.01, ***p < 0.001

We further found, via FACS profiling, that HA-9104 also significantly induced a dose-dependent G2/M arrest in lung cancer cells (Fig. 4a, Supplementary Fig. 4a), which cannot be rescued by NOXA knockdown (Supplementary Fig. 4b), indicating a NOXA independent event. As G2/M arrest is usually induced by DNA damage,^21–23^ we measured the levels of γH2AX upon HA-9104 exposure and found a dose- and time-dependent induction, as revealed by both Western blotting and fluorescent foci assays (Fig. 4b–d, Supplementary Fig. 4c). We then measured what type of DNA damage responses were triggered by HA-9104 by comparing the levels of pATR/pRPA32/pCHK1 and pATM/pCHK2 in non-chromatin and chromatin fractions. HA-9104 treatment caused dose-dependent induction of pATR^T1989^ and pRPA32^S33^, with minimal effect, if any, on pCHK1/pATM/pCHK2 (Fig. 4e, Supplementary Fig. 4d). ATR/RPA32 activation is usually triggered by replication stress,^24,25^ which phosphorylates H2AX as a way to monitor proper DNA replication.^26^

To explore the potential underlying mechanism by which HA-9104 triggered the replication stress, we carefully examined HA-9104 chemical structure and found a 7-azaindole group, which is likely to form the N-H type of hydrogen bonds with thymine base (T), in analogs to adenine base, leading to the possible formation of HA-9104-DNA adduct, thus triggering replication stress (Supplementary Fig. 4e). To test this hypothesis, we examined four HA-9104 derivatives with a series of substitution of trans-3-indoleacrylcamide, for position 4, 5, and 6 of azaindole moiety, designated as compound HA-91011, HA-91012, HA-91013, and with double methyl-substituted indole as compound HA-9101 (Supplementary Fig. 4f). By chemical structures, neither of these derivatives could form two hydrogen bonds with thymine as HA-9104 did, so their potential binding to DNA was expected to be much weaker. Indeed, in comparison to HA-9104, none of these derivatives reduced UBE2F levels, inhibited cullin-5 neddylation, or induced NOXA accumulation as effective as HA-9104. Although to some extent, HA-91011 and HA-91012 induced RPA32^S33^ and H2AX^S139^ phosphorylation in H1650 cells, and HA-91011 also induced G2/M arrest in H358 cells, they were far less effective than HA-9104 (Fig. 4f, g, Supplementary Fig. 4g, h). Thus, it appears that the 7-azaindole group in the HA-9104 structure is responsible for triggering replication stress, γH2AX activation, and G2/M arrest, which is related to UBE2F reduction.

Since replication stress could also trigger apoptosis-like cell death, accompanied by ROS production, which in turn enhances the occurrence of replication stress,^27,28^ we next measured the ROS levels upon HA-9104 exposure to lung cancer cells, and found a significant ROS induction in a dose- and time-dependent manner (Fig. 4h). Consistently, all four HA-9104 derivatives had much less effect on ROS generation (Fig. 4i, Supplementary Fig. 4i). Finally, we found that HA-9104 induced DNA damage irreversibly since γH2AX level remained high upon its withdrawal for up to 24 h (Fig. 4j, Supplementary Fig. 4j). Taken together, it appears that HA-9104 mimics adenine to pair with thymine, thus likely forming the HA-9104-DNA adduct to trigger replication stress and ROS generation, which in turn arrests cells at the G2/M phase as a cellular defensive response.

### HA-9104 suppresses cancer cell growth and sensitizes cancer cells to radiation

**Fig. 5:**
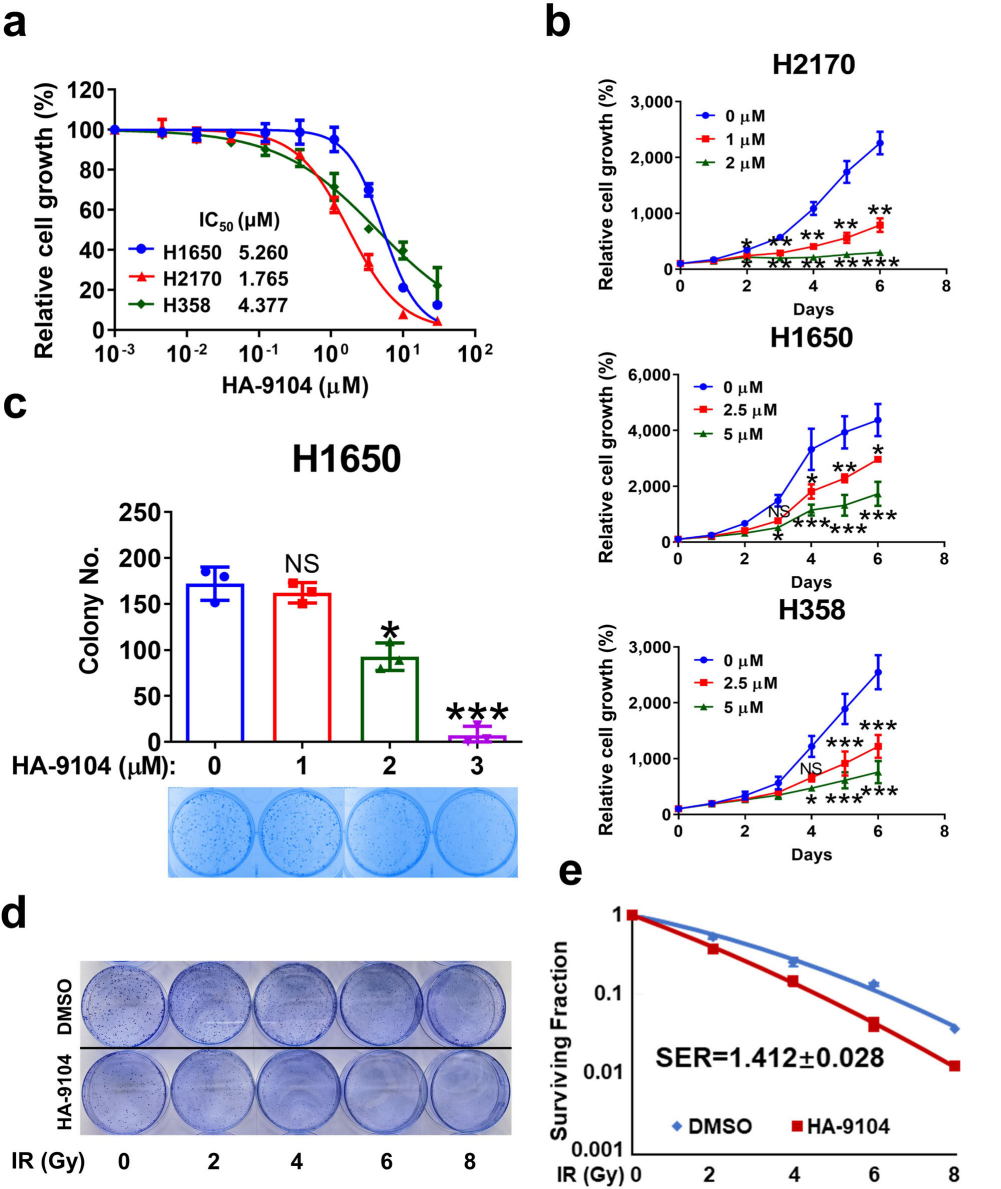
HA-9104 suppresses cancer cell growth and sensitizes cancer cells to radiation. **a** The IC_50_ determination of three lung cancer cell lines. H2170 (5000 per well), H1650 (2500 per well), and H358 (5000 per well) cells were seeded in triplicate in 96-well plates and then treated with various concentrations of HA-9104 for 72 h. Cell viability was detected by ATPlite assays, *n* = 3. **b** The inhibition of tumor cell growth by HA-9104. H2170 (2500 per well), H1650 (1000 per well), and H358 (2500 per well) cells were seeded in triplicate in 96-well plates and then treated with HA-9104 after cell adherence. Cell viability was determined by CCK8 assays, *n* = 3. **c** Colony formation assay. H1650 cells were treated with HA-9104 (0, 1, 2, 3 μM) for 8 days. The data were plotted in a bar graph (up), *n* = 3, and representative dishes were displayed (bottom). **d**, **e** Radiosensitization assay. H1650 cells were seeded into 60 mm dishes and then treated with HA-9104 (1 μM) for 24 h after cell adherence before exposure to radiation. SER was calculated as the ratio of the mean inactivation dose of 1 μM HA-9104 treatment divided by the mean inactivation dose of DMSO treatment, *n* = 3. IR radiation. Shown is mean ± SD. NS not significant, *p < 0.05, **p < 0.01, ***p < 0.001

We next determined the anti-cancer activity of HA-9104 in three lines of lung cancer cells and one pancreatic cancer cell. Using ATPlite-based 72-h cell proliferation assay, HA-9104 showed growth suppression activity with the IC_50_ values ranging from 1 to 5 μM among all cancer cell lines (Fig. 5a, Supplementary Fig. 5a). To increase the solubility of HA-9104, we made three salty forms of HA-9104 (HA-9104-mesylate/**#7**; HA-9104-triflate/**#8**; and HA-9104-hydrochloride/**#9**) (Supplementary Fig. 5b). All salty forms inhibited cullin-5 neddylation, caused NOXA accumulation (Supplementary Fig. 5c) and induced apoptosis (Supplementary Fig. 5d). All had similar or little improved IC_50_ values, as compared to parental HA-9104 (Supplementary Fig. 5e).

We further used a 6-day growth assay to determine the potency of HA-9104 in growth inhibition of lung cancer cells and found an obvious dose-dependent suppression (Fig. 5b). Clonogenic survival assay also demonstrated a dose-dependent suppression of colony formation in H1650 lung cancer cells (Fig. 5c). Note that both H358 and H2170 cells were unable to form colonies under normal culture conditions.

Since HA-9104 induced the G2/M arrest, and cells in the G2/M phases were usually more susceptible to radiation exposure,^29^ and UBE2F had been reported to confer radiation resistance in cancer cells.^30^ We next investigated the radiosensitizing activity of HA-9104. Indeed, HA-9104 significantly sensitized both H1650 and MIAPaCa-2 cells to radiation, with an SER (sensitizing enhancement rate) of 1.41 (Fig. 5d, e) and 1.38, respectively (Supplementary Fig. 5f, g). Thus, HA-9104 is a potent anti-cancer agent with radiosensitizing activity.

### HA-9104 suppresses in vivo tumor growth alone or in combination with radiation

**Fig. 6:**
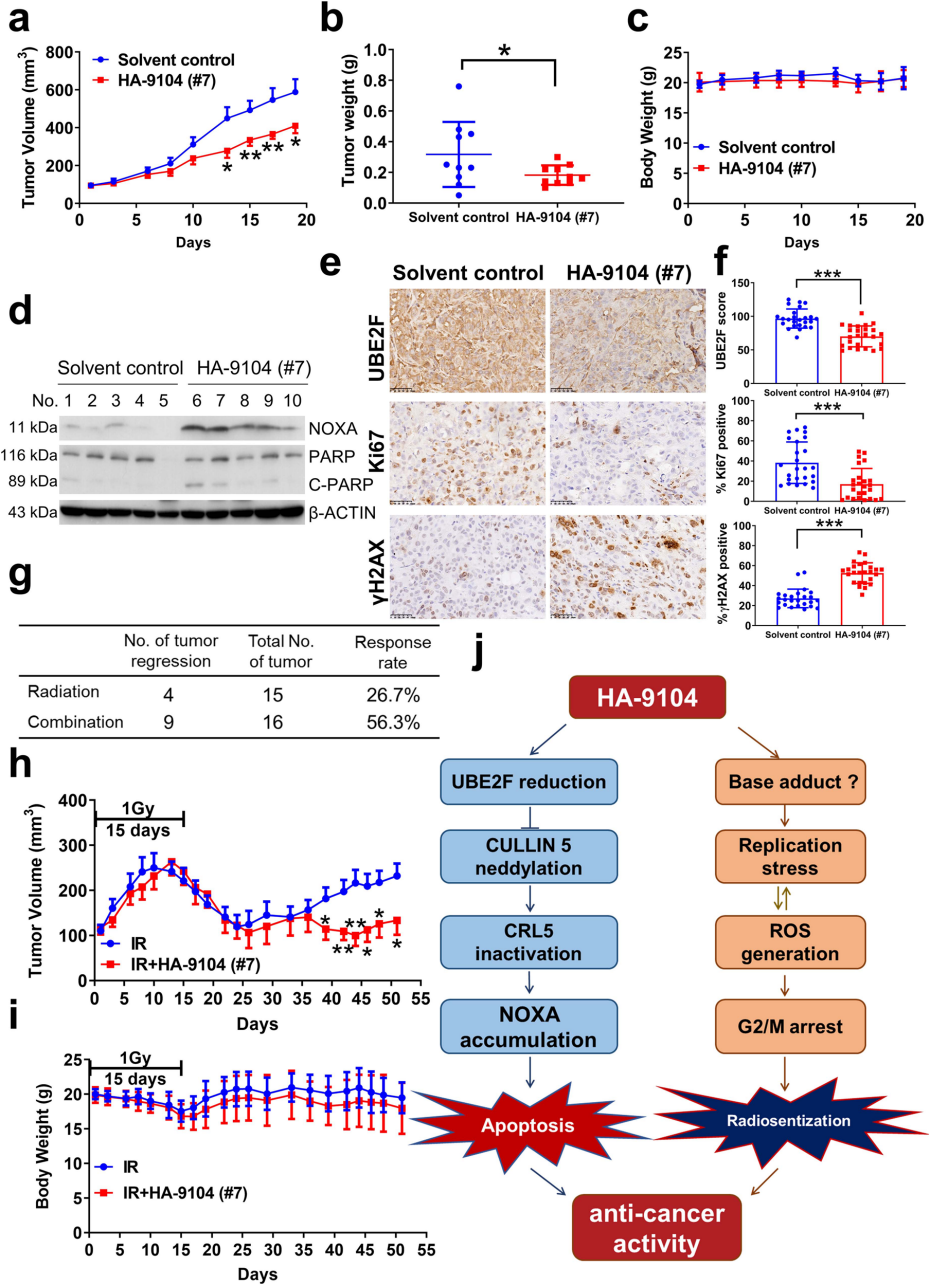
HA-9104 suppresses tumor growth and enhances radiosensitivity in vivo. **a**–**f** In vivo anti-tumor assay. H1650 cells (5 × 10^6^) were injected per flank in both flanks of nude mice. Solvent control or **#7** (30 mg/kg) was injected i.p. when tumor volume reached 80-100 mm^3^ once a day for 19 consecutive days. Tumor volumes (*n* = 10 per group) (**a**), and mice body weight (*n* = 5 per group) (**c**) were measured 2–3 times per week. Tumor weights (*n* = 10 per group) were measured on the last day (**b**). Proteins extracted from tumors were analyzed by Western blotting (**d**) and tumor sections were analyzed using IHC staining. The IHC score was quantified using Image J software, *n* = 5 (**e**, **f**). **g**–**i** In vivo radiosensitization assay. H1650 cells (5 × 10^6^) were injected per flank in both flanks of nude mice. When tumor volume reached 80–100 mm^3^, radiation (1 Gy) was given once a day for consecutive 15 days, 2 h after **#7** (30 mg/kg) was administrated. Tumor response to treatment was summarized (**g**). Tumor volumes (*n* = 15 in radiation alone group, and *n* = 16 in combination group) (**h**), and mice body weight (*n* = 8 per group) (**i**) were measured 2–3 times per week and plotted. IR radiation. **j** Working model. HA-9104, on one hand, binds to UBE2F and reduces UBE2F protein levels, resulting in inactivation of CRL5, accumulation of its substrate NOXA, and thus finally, induction of apoptosis. On the other hand, HA-9104 mimics adenine to trigger replication stress and generate ROS, leading to G2/M arrest and sensitization to radiation. Shown is mean ± SD. **p < 0.01, ***p < 0.001

Finally, we evaluated the in vivo anti-cancer activity of HA-9104 using the nude mice xenograft models. The H1650 cells (5 × 10^6^) were inoculated into both flanks of nude mice. When tumors reached the size of 80–100 mm^3^, tumor-bearing mice were then randomly allocated into solvent control and HA-9104-mesylate (**#7**) treatment groups. The **#7** (30 mg/kg) was administrated via intraperitoneal injection once a day for continuous 19 days. The tumor size was measured 2–3 times every week and a growth curve was plotted. The results showed that **#7** inhibited in vivo tumor growth, as evidenced by reduced tumor size and significantly delayed growth rate (Fig. 6a) and reduced tumor weight (Fig. 6b) without much toxicity as measured by the body weight (Fig. 6c) and H&E staining of important organs (Supplementary Fig. 6a). At the end of the experiment, we selected five tumors from each group randomly and performed both Western blotting and immune-histochemical staining (IHC) to evaluate the in vivo effect of HA-9104. Indeed, the tumor tissues derived from HA-9104-treated mice had increased NOXA levels, enhanced cleavage of PARP (apoptosis) (Fig. 6d), reduced staining of UBE2F (target), Ki67 (proliferation), and increased staining of γH2AX (DNA damage) (Fig. 6e, f).

We also performed in vivo HA-9104 radiosensitization assay with **#7** (30 mg/kg) in combination with radiation (1 Gy per day for continuous 15 days, 2 h post compound dosing), as compared with radiation alone. The combination of **#7** with radiation achieved a greater suppression of tumor growth than radiation alone (Fig. 6g, h, Supplementary Fig. 6b). Specifically, 4 out of 15 tumors in the radiation alone group, whereas 9 out of 16 tumors in the combination group had complete tumor regression. Thus, HA-9104 showed radiosensitization activity in in vivo xenograft lung tumor model. It is worth noting that severe toxicity, as evidenced by greater than 25% loss of body weight, was observed in one mouse in each group. The fluctuation of body weight was shown in Fig. 6i. In general, the combinational group caused slightly more weight loss than the radiation alone group.

## Discussion

It has been more than a decade since the discovery of the first neddylation inhibitor, MLN4924 (Pevonedistat) targeting NAE^16^ with 41 phases I–III clinical trials conducted so far alone or with the combination of several chemotherapeutic drugs (https://clinicaltrials.gov/ct2/results?cond=&term=MLN4924). Many NAE inhibitors have been reported thereafter (for review, see refs. ^31,32^), including one from our group recently.^17^ The most significant one, TAS4464,^33–35^ has completed the phase I clinical trial recently in patients with advanced solid tumors but discontinued due to liver toxicity.^36^ Quite a few small molecular inhibitors targeting DCN1-UBE2M/UBC12 interaction have also been reported (for review, see ref. ^37^), including a recent one that protects mice from liver toxicity induced by acetaminophen.^38^ Finally, our group has recently reported that gossypol has some inhibitory activity against cullin neddylation by targeting the complex of SAG-CUL5 and RBX1-CUL1.^15,32^ However, there is no report on small molecule inhibitors targeting UBE2F so far.

In this study, we report the discovery, through a computer-based virtual screen and structure-based SAR optimization, of HA-9104, as a novel small molecule inhibitor of UBE2F. We provide the following lines of supporting evidence: (1) thermo-stability and thermo-shift assays, as well structure-based docking assay showed that HA-9104 binds to UBE2F likely on the N152 and/or V98 pockets; (2) enzyme-based in vitro assays demonstrated that HA-9104 abrogates UBE2F~NEDD8 thioester formation and inhibits cullin-5 neddylation; and (3) cell-based in vitro assays showed that HA-9104 reduces UBE2F protein levels, and selectively inhibits cullin-5 neddylation to inactivate CRL5 activity and cause NOXA accumulation. To the best of our knowledge, HA-9104 is the first small molecule inhibitor reported to target UBE2F. It is worth noting that our virtual screening was in an attempt to identify small molecules that disrupt the UBE2F-UBA3 binding on the V30 pocket. However, the structure-based docking simulation, and thermo-stability and thermo-shift assays of UBE2F mutants suggest that HA-9104 is likely targeting N152 and/or V98 pockets, leading to altered protein stability and loss of enzymatic activity.

One unsolved puzzling issue in our study is how HA-9104 causes a reduction in UBE2F protein levels. HA-9104 did not change the levels of UBE2F mRNA (Fig. 2c, Supplementary Fig. 2c), excluding possible regulation at the transcriptional levels; failed to alter the rate of UBE2F translation (Fig. 2g, Supplementary Fig. 2h), excluding possible regulation at the translational levels. Furthermore, the failure in blockage of HA-9104-induced UBE2F reduction by proteasome inhibitor MG132 or lysosome inhibitor CQ (Fig. 2e, Supplementary Fig. 2f) excluded the possible regulation at the post-translational levels through the degradation by UPS or lysosome system. Nevertheless, we found that the effect of HA-9104 on UBE2F is rather specific since its several structural derivatives failed to reduce UBE2F levels (Fig. 4f, Supplementary Fig. 4g). Future investigation will be geared to elucidate the underlying mechanism of HA-9104 action, such as promoting UBE2F proteolysis.

It is worth noting that although the ribosomal profiling assay did not reveal inhibition of UBE2F translation (Fig. 2g, Supplementary Fig. 2h), HA-9104, however, did moderately increase the 80S monosome fraction and reduced polysome fractions (Fig. 2f, Supplementary Fig. 2g), indicative of stalled translation initiation and reduced ribosomal translation process in general. However, HA-9104 is not a general inhibitor of protein translation since the mass spectrometry-based proteomic analysis detected only 46 proteins with a twofold reduction upon HA-9104 treatment (Supplementary Fig. 2i) (also in PXD036191). It is reasonable to speculate that replication stress and ROS generation induced by HA-9104, could impair the mRNA translation to some extent through oxidation of the cysteine on translational regulatory proteins.^39,40^ The detailed involving mechanism is an interesting project for future investigation.

It is known that ATR activation converges a variety of the replication stress responses through its major downstream effector kinase CHK1 for replication restart and DNA repair.^21,22,41,42^ Interestingly, while ATR activation did cause RPA32 activation to trigger the replication stress response, it failed to activate CHK1 via Ser345 phosphorylation, implying a failure in replication restart and DNA repair. This is indeed consistent with the observation that HA-9104 caused irreversible DNA damage, as evidenced by the failure in the recovery of elevated γH2AX levels back to the basal levels even 24 h after compound removal (Fig. 4j, Supplementary Fig. 4j). We acknowledge that we did not provide experimental evidence to show that HA-9104 directly interacts with DNA, thereby inducing DNA damage. We have attempted to biotin-labeling HA-9104 and its precursor **iV26-9** (Fig. S1C). Unfortunately, attachment of biotin to the piperazine group of **iV26-9** or 7-azaindole group of HA-9104 abrogated their activities in (1) reducing UBE2F protein levels, (2) blocking cullin-5 neddylation and inducing NOXA accumulation, and (3) inducing DNA damage. Thus, we were unable to label HA-9104 with a fluorescent dye or similar molecule for a direct DNA binding assay.

HA-9104 displayed sound activity in the suppression of growth and survival of lung cancer cells in in vitro cell-based assays, with the IC_50_ values ranging around 1–5 μM. However, the in vivo anti-tumor activity in xenograft tumor models is not very substantial, largely due to two reasons: (1) HA-9104 has a poor solubility even in salty form, and (2) HA-9104 has a very short half-life of 6 min, as measured by an in vitro liver microsomal metabolic stability assay (Supplementary Fig. 5h). The emerging biomaterials, like liposomes, albumin nanoparticles and polymeric micelles^43–45^ may overcome these shortages to some extent, but ultimate improvement relies on thorough SAR optimization to make it oral available with much better pharmacokinetics. Finally, our study, using both in vitro and in vivo tumor models, demonstrated that HA-9104 has sound radiosensitizing activity in both lung and pancreatic cancer cells. This effect is likely attributable to its activity in inducing apoptosis via NOXA accumulation and G2/M arrest via replication stress and ROS generation.

In summary, we proposed the following working model in which HA-9104 acts as a novel class of anti-cancer small molecule. HA-9104, on one hand, reduces UBE2F levels (via a yet-to-defined mechanism) to inhibit cullin-5 neddylation, resulting in CRL5 inhibition and NOXA accumulation to trigger apoptosis. HA-9104, on the other hand, causes DNA base adduct (most likely) to activate ATR/RPA32 and replication stress, ROS generation, and G2/M arrest for radiosensitization (Fig. 6j).

## Materials and methods

### Animal experiments

Animal experiments were approved by the Animal Ethics Committee of Zhejiang University; animal care was provided under the principles and procedures of the regulatory standards at Zhejiang University Laboratory Animal Center.

### Cell culture

Human lung cancer cell lines H2170, H1650, H358, and human pancreatic cancer cell line MIAPaCa-2 were obtained from American Type Culture Collection (ATCC). The culture medium RPMI 1640 was used for H2170, H1650, and H358 cells, whereas DMEM was used for MIAPaCa-2, both containing 10% fetal bovine serum and 1% penicillin/streptomycin. All cells were incubated at 5% CO_2_, 37 °C, with 95% humidity.

### Chemicals

MLN4924 was from ApexBio (#B1036). Chlorhexidine (CHX) was from Sigma-Aldrich (#C7698), whereas hydroxyurea (HU) was from MedChem Express (HY-B0313). SYPRO orange was from Sigma-Aldrich (S5692). The iFluor 680 was from ATT Bioquest (1240).

### Protein purification

The plasmids encoding human SAG-CUL5, UBE2F, UBA3/APPBP1, and NEDD8 were constructed and purified as described previously.^15^ UBE2F-wt and three UBE2F mutants (V30, V98, and N152) were cloned into the pET-28a vector with a SUMO-tag by Tsingke Biotech (Beijing, China), expressed in Rosetta 2(DE3) pLysS (Novagen). The proteins were purified by Ni-NTA agarose beads (QIAGEN) with His-SUMO tags cleaved by the His-tagged Ulp1 enzyme. The protein mixture was incubated with Ni-NTA agarose beads which bind His-Ulp1 and the cleaved His-SUMO tags, and un-tagged recombinant protein was collected as flow-through, and stored in wash buffer (25 mM HEPES pH 7.5–7.8 and 50 mM NaCl) at −80 °C.

### Western blotting and antibodies

Cells were treated with various compounds, and the protein levels were measured by standard Western blotting analysis. The antibodies used were obtained from the following vendors: CUL-5 (sc-373822, Santa Cruz/SC), CUL-1 (sc-11384, SC), CUL-2 (ab166917, Abcam), CUL-3 (2759S, Cell Signaling Technology/CST), CUL-4A (2699S, CST), CUL-4B (12916-1-AP, Proteintech), NOXA (OP180, EMD Millipore), UBE2F (sc-398668, SC), UBE2M (sc-390064, SC), PARP (9542S, CST), Caspase 3 (9662S, CST), CUL-5 CTD (AV35127, Sigma-Aldrich), CUL-1 CTD (12895-1-AP, Proteintech), NEDD8 (ab81264, Abcam), p-ATR T1989 (2853S, CST), ATR (2790S CST) p-RPA32 S33 (ab211877, Abcam), RPA32 (ab2175, Abcam), p-CHK1 S345 (2348, CST), CHK1 (sc-8408, SC), p-ATM S1981 (5883S, CST), ATM (2873S, CST), p-CHK2 T68 (2197P, CST), CHK2 (6334S, CST), γH2AX (05-636, EMD Millipore), DAPI (D1306, Invitrogen), β-Actin (M1210-2, HuaBio) and α-Tubulin (T8203, Sigma-Aldrich).

### Docking simulations

All docking simulations were carried out by the *Glide* module in Schrödinger 9.0,^46^ with the crystal structure of human UBE2F (PDB entry: 3FN1),^5^ as described^15^ with the maximum root-mean-square deviation value setting to 0.3 Å, and the scaling factors for van der Waals radii and partial atomic charge cutoff value setting to 0.8 and 0.15, respectively. The structure of HA-9104 was prepared by the *LigPrep* module with protonated states generated at pH = 7.0 ± 2.0 with all other parameters set to the default values. HA-9104 was docked into the structure of UBE2F, and the standard precision was used to score and rank the binding affinities.

### The in vitro thermal shift assay (TSA)

The assays were performed as described recently.^15^ Briefly, purified UBE2F (at a final concentration of 0.4 μg/mL) was incubated with 20 μM HA-9104-mesylate, along with DMSO control, in 50 μL reaction buffer at 25 °C for 10 min, and then heated at various temperatures for 5 min. UBE2F protein levels were measured by Western blotting.

### The in vitro fluorescent thermal shift assay

The melting temperature (Tm) values were measured using an in vitro fluorescent thermal shift assay (or differential scanning fluorimetry, DSF) as previously described.^18,19^ Briefly, 2 μM pure UBE2F was incubated with indicated concentrations of HA-9104-mesylate mixed with SYPRO orange, and heated to a temperature gradient of 1 °C/min from 25 to 95 °C. The fluorescence was monitored in an RT-PCR machine (*Applied Biosystems StepOne*^TM^) with the ROX filters.

### Biochemical assays

#### The in vitro E1 NEDD8 thioester assay

The assays were performed as described recently.^15^ Briefly, purified E1 (UBA3/APPBP1, 50 nM) and NEDD8 (200 nM) were incubated with indicated compounds (final DMSO 1%) in a reaction buffer at 4 °C for 10 min. The reaction was initiated by the addition of 200 μM ATP, incubated at 16 °C for 10 min, followed by quenching with SDS loading buffer and Western blotting.

#### The in vitro E2 NEDD8 thioester assay

The assays were performed as previously described.^15^ Briefly, purified NEDD8 protein was labeled with iFluor 680 dye and designated as iNEDD8. The reaction in a buffer, containing 200 nM iNEDD8, 50 nM UBA3/APPBP1, and 50 nM UBE2F, was initiated by the addition of 200 μM ATP after a 10-min pre-incubation with HA-9104 at various concentrations. The reaction was quenched after 10 min of incubation at 25 °C by adding SDS loading buffer, subjected to SDS–PAGE gel, and detected by an *Odyssey* two-color infrared laser imaging system (LI-COR, USA).

#### The in vitro cullin neddylation assay

The assays were performed as previously described.^15^ Briefly, the reaction in a buffer, containing 300 nM NEDD8, 25 nM UBA3/APPBP1, 200 nM UBE2F, and 200 nM SAG-Cullin 5 E3 complex (SAG-CUL5^CTD^) was initiated by the addition of 200 μM ATP after a 20-min pre-incubation with HA-9104 at various concentrations. The reaction was quenched after 15 min of incubation at 25 °C by adding SDS loading buffer, followed by Western blotting.

#### The in vitro cullin neddylation assay (Coomassie brilliant blue staining)

The assays were performed as previously described.^15^ Briefly, the reaction in a buffer, containing 3 μM NEDD8, 50 nM UBA3/APPBP1, 1 μM UBE2F (wt or its V98 and N152 mutants), and 1 μM SAG-CUL5^CTD^ was initiated by the addition of 200 μM ATP. The reaction was quenched after indicated incubation at 25 °C by adding SDS loading buffer, followed by SDS-PAGE gel separating and Coomassie brilliant blue staining.

### Quantitative real-time reverse-transcription PCR (qRT-PCR)

The assays were performed as described recently.^17^ The primers used for qRT-PCR were as follows:5′-GAC TGT TCG TGT TCA GCT CG-3′ and 5′-CAC TCG ACT TCC AGC TCT GCT-3′ for NOXA; 5′-GAC CGG GCA TGG TGT TGG-3′ and 5′-ACC ATC GTC ACG CTT CAG TT-3′ for UBE2F; 5′-GGA GTC AAC GGA TTT GGT-3′ and 5′-GTG ATG GGA TTT CCA TTG AT-3′ for GAPDH as an internal control.

### Ribosome profiling

The assays were performed as recently described,^17^ using an Auto Gradient Fractionator (Biocomp, Canada). The total RNA was isolated from collected fractions (600 μL/per fraction), followed by the synthesis of cDNAs and qRT-PCR.

### Quantitative proteomic analysis

H2170 cells were treated with DMSO or HA-9104 (10 μM) for 24 h in duplicates, then washed with ice-cold PBS 3 times, and harvested for quantitative proteomic analyses by Jingjie PTM Biolab Co, Ltd. (Hangzhou, China). The resulting MS/MS data were processed and analyzed using the MaxQuant search engine (v.1.6.15.0).

A twofold change threshold, and CV (coefficient of variation) < 0.1 was set as the cut-off in which the difference after HA-9104 treatment was considered significant.

### Half-life determination

Cells were treated with HA-9104 in the presence of CHX (50 μg/mL) for various time points, followed by Western blotting and densitometry quantification via ImageJ software (NIH).

### In vivo ubiquitination assay

The assays were performed as previously described.^47^ Briefly, cells were transfected with His-tagged ubiquitin for 24 h, then treated with HA-9104 (10 μM), along with DMSO control for another 24 h. MG132 (20 μM) was added to the medium 6 h before harvest. Cells were lysed using a 6 M guanidinium denaturing buffer. An equal amount of whole cell lysate was incubated with Ni-NTA beads at room temperature for 4 h. Beads were then washed with a series of buffers, as previously described.^47^ Proteins were then eluted from beads, and subjected to Western blotting for NOXA polyubiquitination with the anti-NOXA antibody.

### Apoptosis

Cells were treated with HA-9104, stained with *Annexin V*-*FITC* staining kit (BD Pharmingen, Germany), and then analyzed by a CytoFLEX S flow cytometer (Beckman Coulter, USA).

### Cell cycle

Cells after HA-9104 treatment were fixed in ice-cold 70% ethanol at 4 °C overnight and suspended in 500 μL PI/RNase staining buffer (BD Pharmingen, USA) for 15 min in the dark after 3 × PBS washing. The samples were analyzed by a CytoFLEX S flow cytometer for cell cycle profiling.

### Intracellular ROS detection

ROS was measured using dichlorofluorescin diacetate (DCFH-DA) reagent (Beyotime, China) according to the manufacturer’s instructions. Briefly, cells after HA-9104 treatment were washed with serum-free medium, incubated with DCFH-DA at 37 °C for 20 min, followed by 3 × PBS washing. The levels of ROS were detected by a CytoFLEX S flow cytometer.

### DNA fragmentation analysis

The assays were performed as previously described.^48^ Briefly, cells after HA-9104 treatment were lysed in a lysis buffer. Genomic DNA was isolated and subjected to a 1.8% agarose gel (Invitrogen), and photographed by Gel Doc XR + System (Bio-Rad, USA).

### Transfection of siRNAs

The siRNA transfection was conducted in lung cancer cells with the GenMute siRNA Transfection Reagent (SignaGen Laboratories, USA). The siRNA sequences were as follows: *siNOXA: UGC ACG UUU CAU CAA UUU GTT;* and *siCont: UUC UCC GAA CGU GUC ACG UTT.*

### Immunofluorescence

Cells after indicated treatment were fixed with ice-cold methanol (30 min at −20 °C), followed by 3× PBS washing, and then stained with anti-γH2AX Ab and DAPI. Cells were then photographed with a Nikon A1 Ti confocal microscope (Nikon, Japan).

### Chromatin protein extraction

Cells after indicated treatment were lysed in lysis I buffer (50 mM HEPES, pH = 7.5, 1 mM EDTA, 150 mM NaCl, 0.1% Triton X-100 (v/v), 1 mM PMSF and 1% protease inhibitor cocktail) on ice. After centrifugation, the supernatant was collected as non-chromatin protein. The pellet was dissolved in lysis II (10 mM Tris-HCl, pH = 7.5, 5% SDS (w/v), and 1% protease inhibitor) with sonication and used as chromatin fraction.

### IC_50_ determination

Cells were seeded in 96-well plates in triplicate and treated with HA-9104 at various concentrations for 72 h. Cell growth was assayed by Cell Counting Kit-8 (CCK-8) (MedChem Express) at OD_450_ in a microplate reader (SpectraMax iD3, Molecular Devices), or by the ATPlite 1 step Luminescence Assay System (PerkinElmer) in a microplate reader. The inhibition curve was drawn by GraphPad Prism software.

### Radiation exposure and clonogenic assay

Cells were seeded in 60 mm dishes in duplicate and treated with HA-9104 for 24 h after adherence. Cells were then exposed to radiation (X-RAD 160; Precision X-Ray, Inc., Kentwood), cultured at 37 °C for another 7 days, and stained with Coomassie brilliant blue solution. Colonies with greater than 50 cells were counted under an inverted microscope. Survival curves were generated as described.^49^ The radiosensitization assay with a calculation of sensitizing enhancement rate (SER) was performed as described.^50^

For the clonogenic assay, H1650 cells were seeded 600 per well in six-well plates in triplicate, followed by incubation at 37 °C for another 8 days with HA-9104 (1, 2, 3 μM). The colonies were then stained and counted.

### The in vivo anti-tumor assay

H1650 cells (5 × 10^6^ cells suspended in 100 μL PBS per tumor) were inoculated subcutaneously in both franks into female BALB/C nude mice (5–6 weeks old) (Shanghai Slac laboratory animal Co., Ltd., China). The mice were randomized into control or experimental groups when the tumor size reached approximately 80–100 mm^3^. **#7** in DMSO (30 mg/kg) or DMSO vehicle was administrated to mice by intraperitoneal injection, once a day for 19 consecutive days. For combination treatment, radiation (1 Gy) was given once a day for 15 consecutive days, two hours post **#7** injection. Tumor growth/tumor size and body weight were monitored 2–3 times a week, and average tumor volumes were calculated by the formula (*L* × *W*^2^)/2. At the end of the experiment, tumors were harvested, weighed, and photographed. We used a humane protocol in our mouse xenograft tumor growth assay with the endpoints of tumor volume <1500 mm^3^.

### Statistical analysis

The statistical analysis was assessed using GraphPad Prism software v 7.0 (San Diego, CA. USA). The student’s t-test was used for the comparison of parameters between groups. Three levels of significance (*p < 0.05, **p < 0.01, ***p < 0.001) were presented.

## Supplementary information

Supplementary Figure

## Data Availability

All data and methods in this study are available upon request. The mass spectrometry proteomics data have been deposited to the ProteomeXchange Consortium via the PRIDE partner repository (http://www.proteomexchange.org) with the dataset identifier No. PXD036191.
