# Supplementary material for: A small molecule inhibitor of the UBE2F-CRL5 axis induces apoptosis and radiosensitization in lung cancer
Source: Signal Transduct Target Ther. 2022 Oct 17;7:354. doi: 10.1038/s41392-022-01182-w (PMC9576757; doi:10.1038/s41392-022-01182-w)
Supplement: Supplementary file 1 — Supplementary Figure [file 41392_2022_1182_MOESM1_ESM.docx]

Supplementary Materials for

**A small molecule inhibitor of the UBE2F-CRL5 axis induces apoptosis and radiosensitization in lung cancer**

Tiantian Xu^1-3, #^, Qisheng Ma^4, #^, Yanan Li^1,2^, Qing Yu^1,2^, Peichen Pan^5^, Yawen Zheng^6^, Zhijian Li^1-3^, Xiufang Xiong^1-3^, Tingjun Hou^5^, Bin Yu^4^, Hongmin Liu^4^,

and Yi Sun^1-3, *^

Correspondence to: yisun@zju.edu.cn

**This PDF file includes:**

Figures. S1 to S6

**
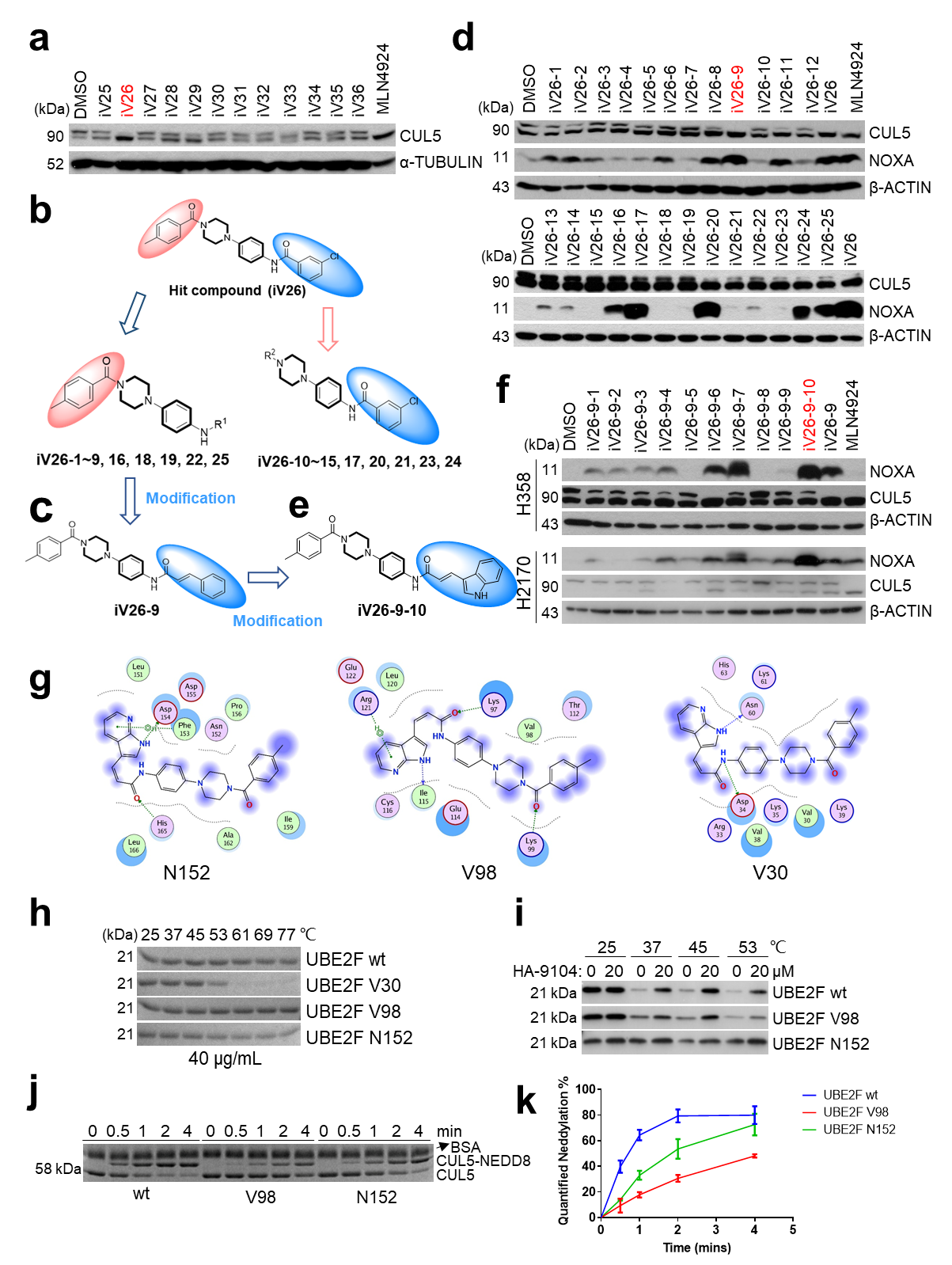
**

**Figure. S1 Discovery of HA-9104, which stabilizes UBE2F *in vitro.***

**a-f** Structure-activity relationship-based optimization, from **iV26** **b** to **iV26-9** **c** to **iV26-9-10** **e.** Representative Western blotting results of indicated compounds **a, d, f**. H358 and H2170 cells were treated with indicated molecules (20 μM) for 24 hours, followed by Western blotting. **g** 2-D schematic diagram of the binding patterns of HA-9104 to UBE2F in different regions with the hydrogen bonds highlighted. Color codes: the pink (polar amino acids); the green (greasy residues). **h** Comparison of thermo-stability of UBE2F-wt with its three mutants. Purified UBE2F-wt or three mutants (40 μg/mL) in 50 μL buffer was heated at the indicated temperature for 5 minutes. UBE2F protein levels were measured by Coomassie brilliant blue staining. **i** Comparison of thermo-stability of UBE2F-wt with V98 and N152 mutants upon exposure to HA-9104. Purified UBE2F wt or two mutants (0.4 μg/mL) was incubated individually with HA-9104 (20 μM) at 25℃ for 10 minutes in 50 μL buffer and then heated at the indicated temperature for 5 minutes. UBE2F protein levels were measured by Western blotting. **j-k** Comparison of enzymatic activity of UBE2F-wt with V98 and N152 mutants in *in vitro* cullin-5 neddylation assay. The reaction mixture, containing 3 μM NEDD8, 50 nM UBA3/APPBP1, 1 μM UBE2F, and 1 μM SAG-CUL5 E3 complex (SAG-CUL5^CTD^) was initiated by the addition of 200 μM ATP. The reaction was quenched after indicated incubation at 25℃ by adding SDS loading buffer, followed by SDS-PAGE gel separating and Coomassie brilliant blue staining **j.** The rate of cullin-5 neddylation was quantified by Image J and plotted. Shown is mean ± SD from three independent assays **k**.


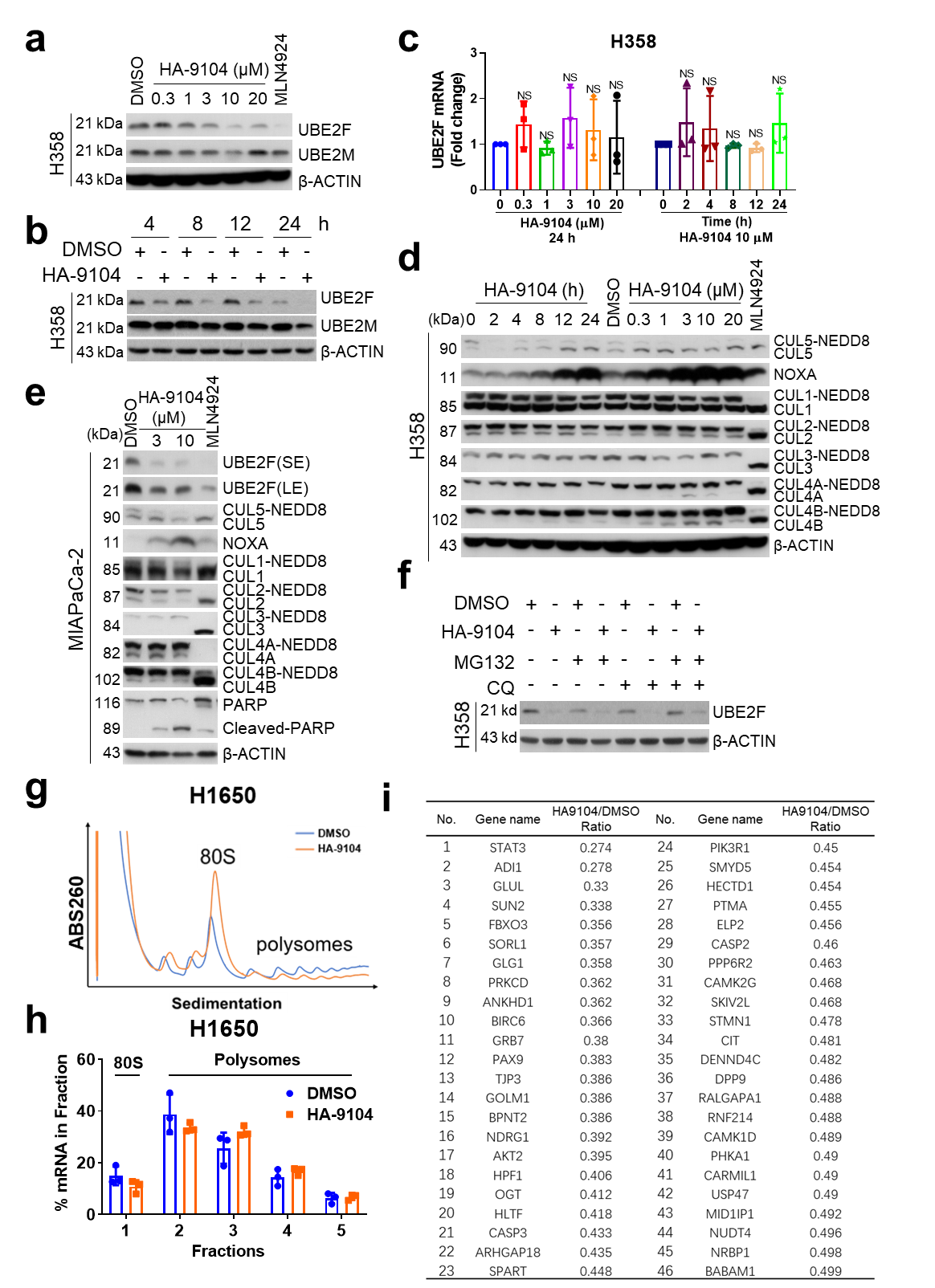


**Figure. S2 HA-9104 reduces UBE2F protein levels**

**a-b** HA-9104 reduces the levels of UBE2F protein. H358 cells were treated with HA-9104 at various concentrations for 24 hours, or MLN4924 (0.3 μM) as a positive control **a**, or treated with DMSO or HA-9104 (10 μM) for indicated time periods **b**, and then harvested for Western blotting. **c** HA-9104 treatment did not affect UBE2F mRNA levels. H358 cells were treated with HA-9104 at various concentrations for 24 hours (left), or treated with HA-9104 (10 μM) for indicated time periods (right), followed by qRT-PCR, n=3. **d-e** HA-9104 preferentially inhibits cullin-5 neddylation and induces its substrate NOXA accumulation. H358 cells **d** and MIAPaCa-2 cells **e** were treated with HA-9104 for indicated time periods and then harvested for Western blotting. SE, shorter exposure; LE, longer exposure. **f** HA-9104 did not affect UBE2F turnover. H358 cells were treated with HA-9104 (10 μM) or DMSO for 24 hours. MG132 (10 μM) or/and CQ (50 μM) were added 6 hours before harvest, followed by Western blotting. **g-h** HA-9104 inhibits global translation, but not UBE2F translation. H1650 cells were treated with DMSO or HA-9104 (10 μM) for 24 hours, then subjected to ribosome profiling. **g** A representative curve of mRNA translation. **h** The qRT-PCR result of UBE2F mRNA, n=3. **i** A list of proteins with 2-fold reduction by HA-9104 treatment in H2170 cells through quantitative proteomic analyses (PXD036191). H2170 cells in duplicates were treated with DMSO or HA-9104 (10 μM) for 24 hours, then subjected to quantitative proteomic analysis. Shown is mean ± SD. NS: not significant.


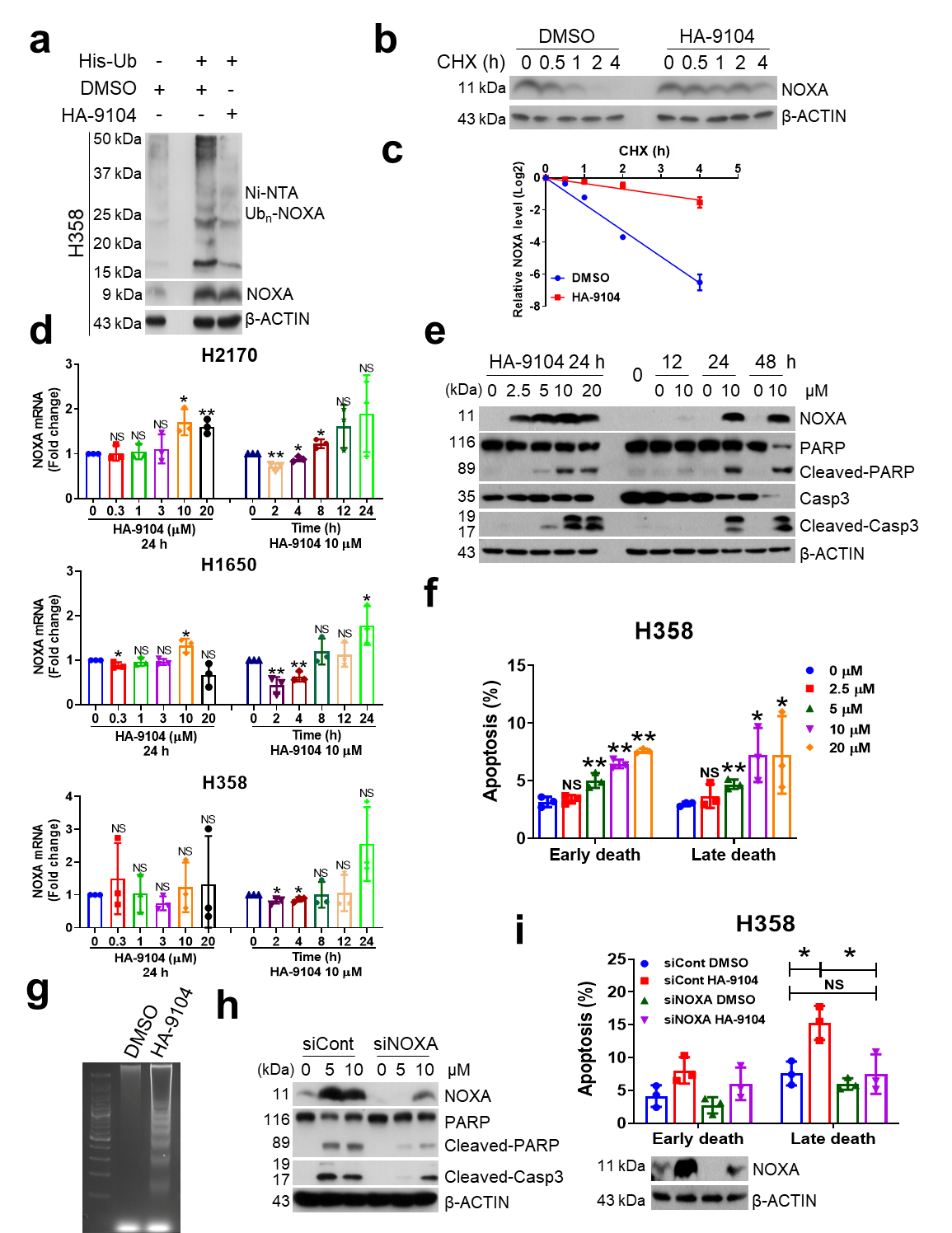


**Figure. S3 HA-9104 causes NOXA accumulation to induce apoptosis.**

**a** HA-9104 inhibits NOXA polyubiquitination. H358 cells were transfected with plasmid encoding His-tagged ubiquitin for 24 hours, then treated with HA-9104 (10 μM), along with DMSO control for another 24 hours. Cell lysates were subjected to *in vivo* ubiquitylation assay as described in M&M. **b-c** HA-9104 prolongs NOXA protein half-life. H358 cells were treated with HA-9104 (10 μM) or DMSO control in the presence of CHX (50 μg/mL) for indicated time periods, followed by Western blotting. The band density was quantified by Image J software, n=3. **d** HA-9104 treatment has a limited effect on NOXA mRNA level. H2170, H1650, and H358 cells were treated with HA-9104 at various concentrations for 24 hours (left), or treated with HA-9104 (10 μM) for indicated time periods (right), followed by qRT-PCR, n=3. **e-g** HA-9104 induces apoptosis in a dose- and time-dependent manner. H358 cells were treated with various concentrations of HA-9104 for 24 hours (left), or with HA-9104 (10 μM) for indicated time periods (right), and then subjected to Western blotting **e**, combined annexin-V and PI staining **f**, and DNA fragmentation assay **g**. **h-i** Silencing of NOXA partially rescues apoptosis induced by HA-9104. H358 cells were transfected with siCont or siNOXA for 48 hours and then treated with HA-9104 (5, 10 μM) or DMSO control for another 24 hours, followed by Western blotting **h** or combined annexin-V and PI staining **i**. Shown is mean ± SD. NS: not significant, *p < 0.05, **p < 0.01.


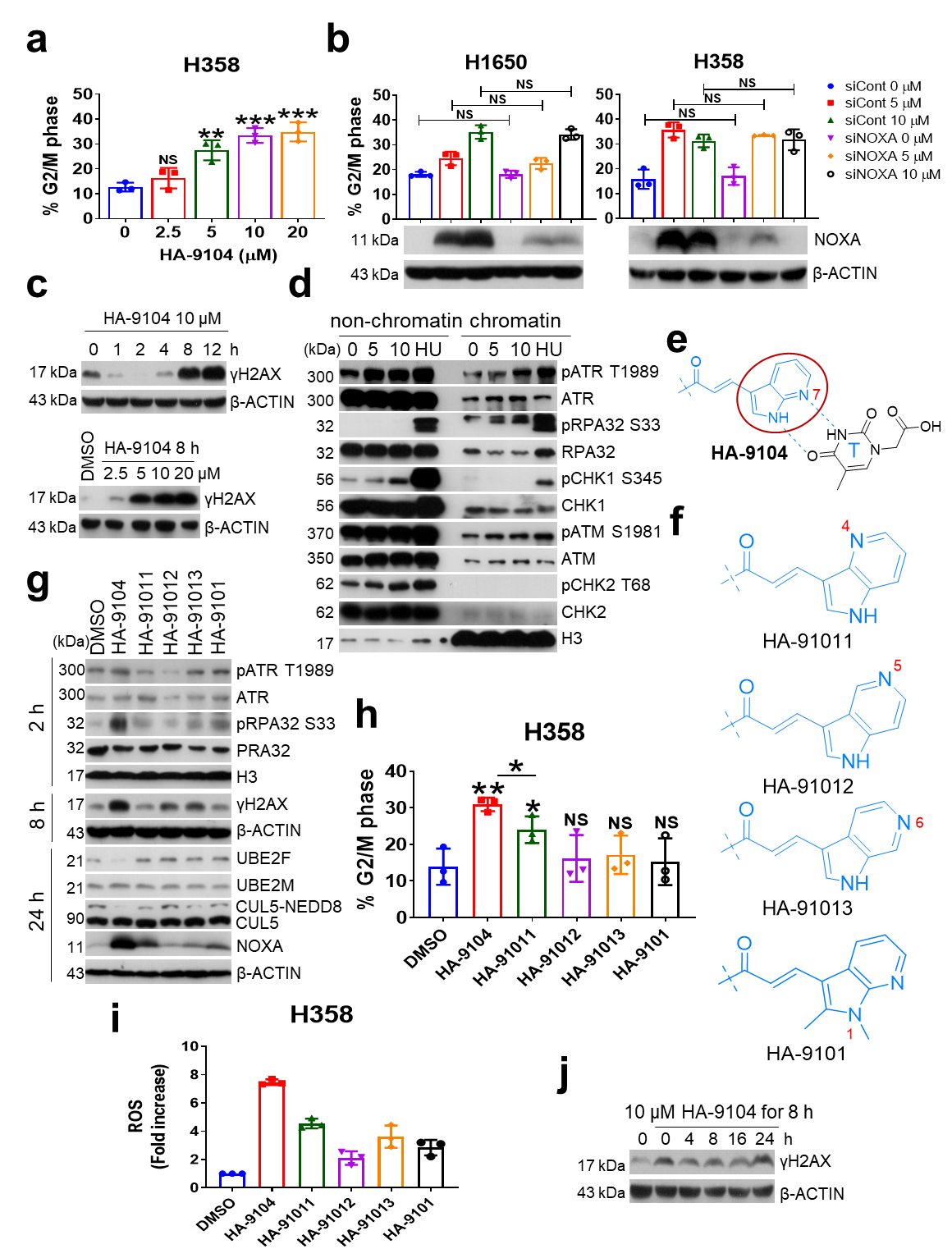


**Figure. S4 HA-9104 induces DNA damage to trigger G2/M arrest.**

**a** HA-9104 causes G2/M arrest. H358 cells were treated with various concentrations of HA-9104 for 24 hours, followed by FACS analysis. The histogram of cell percentage was shown, n=3. **b** HA-9104 caused G2/M arrest is independent of NOXA. H1650 and H358 cells were transfected with siCont or siNOXA for 48 hours and then treated with HA-9104 (5,10 μM) for another 24 hours, followed by FACS analysis. The histogram of cell percentage was shown, n=3. **c** HA-9104 causes γH2AX accumulation. H358 cells were treated with HA-9104 (10 μM) for indicated time periods (up) or indicated concentrations of HA-9104 for 8 hours (down), and then harvested for Western blotting. **d** HA-9104 treatment activates the ATR pathway. H358 cells were treated with HA-9104 (5, 10 μM) for 2 hours. HU (5 mM) was used as a positive control. Chromatin proteins were isolated from whole cell lysate, followed by Western blotting. **e** Schematic diagram of HA-9104 pairing with thymine. **f** The structures of HA-9104 derivatives. **g-i** Comparison of HA-9104 with its derivatives. H358 cells were treated with indicated drugs (10 μM) for indicated time periods and then analyzed for the activation of the ATR pathway (2 hours), γH2AX induction (8 hours), UBE2F reduction and CRL5 inactivation (24 hours) **g**, G2/M arrest (24 hours), n=3 **h**, and ROS generation using DCFH-DA labeling (6 hours), n=3 **i**. **j** HA-9104 induces irreversible DNA damage. H358 cells were treated with HA-9104 (10 μM) for 8 hours first, and then washed with PBS 3 times and replaced with fresh medium without HA-9104 for indicated time periods, followed by Western blotting. Shown is mean ± SD. NS: not significant, *p < 0.05, **p < 0.01, ***p < 0.001.

**
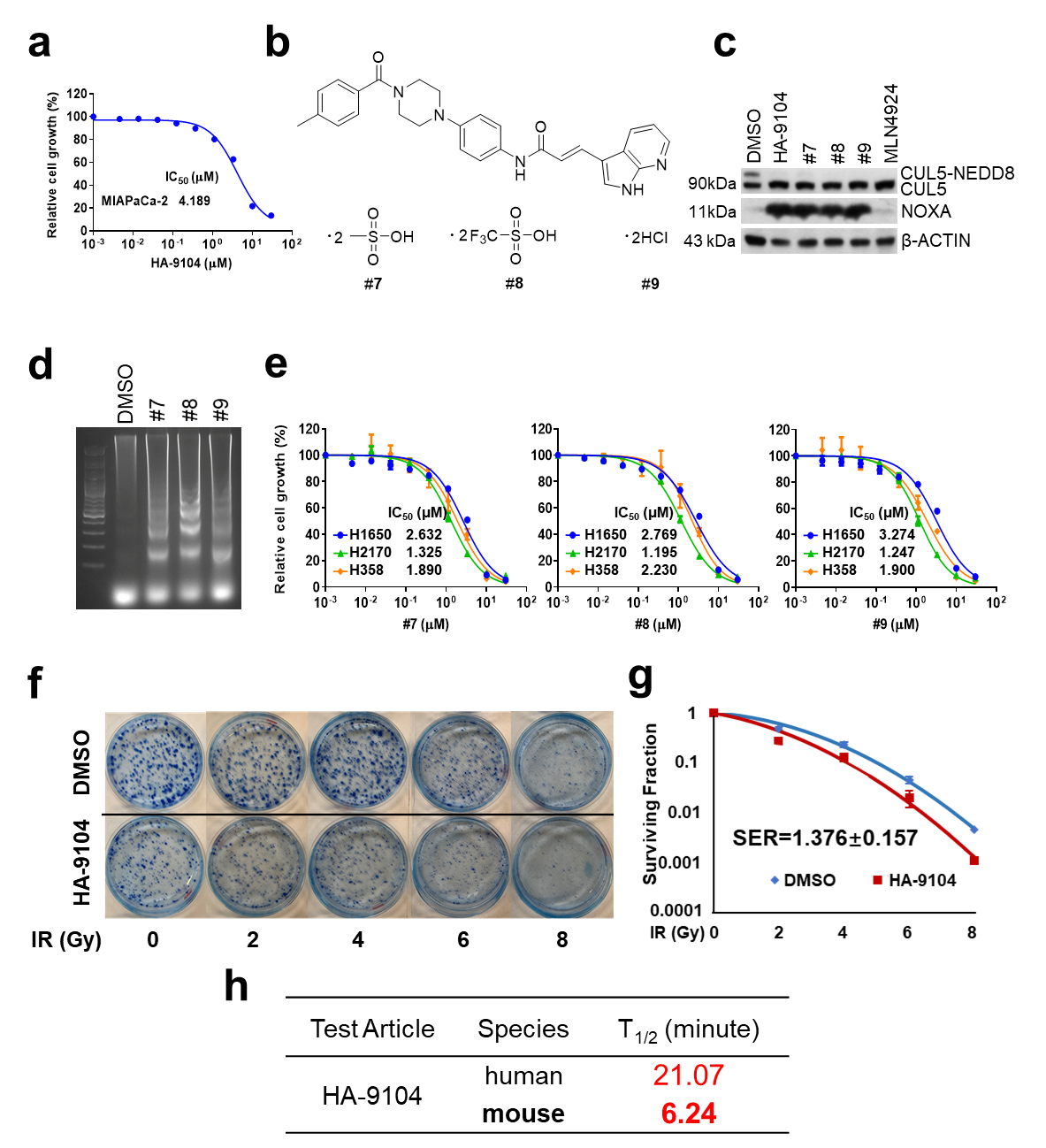
**

**Figure. S5 HA-9104 suppresses cancer cell growth and sensitizes cancer cells to radiation.**

**a** IC_50_ determination. MIAPaCa-2 cells were seeded in triplicates in a 96-well plate and then treated with various concentrations of HA-9104 for 72 hours. Cell viability was detected by ATPlite assays, n=3. **b-e** The structures of three salty forms of HA-9104 **b**, and their effects on the cullin-5 neddylation inhibition, NOXA induction **c**, apoptosis induction **d**, and cell growth inhibition **e**. **f-g** Radiosensitization assay. MIAPaCa-2 cells were seeded into 60 mm dishes and then treated with HA-9104 (0.3 μM) for 24 hours after cell adherence before radiation exposure. Sensitizing enhancement rate (SER) was calculated as the ratio of the average inactivation dose of HA-9104 treatment divided by the average inactivation dose of DMSO treatment, n=3. IR, radiation. **h** Liver microsomal metabolic stability. HA-9104 was subjected to the test. Shown is mean ± SD.

**
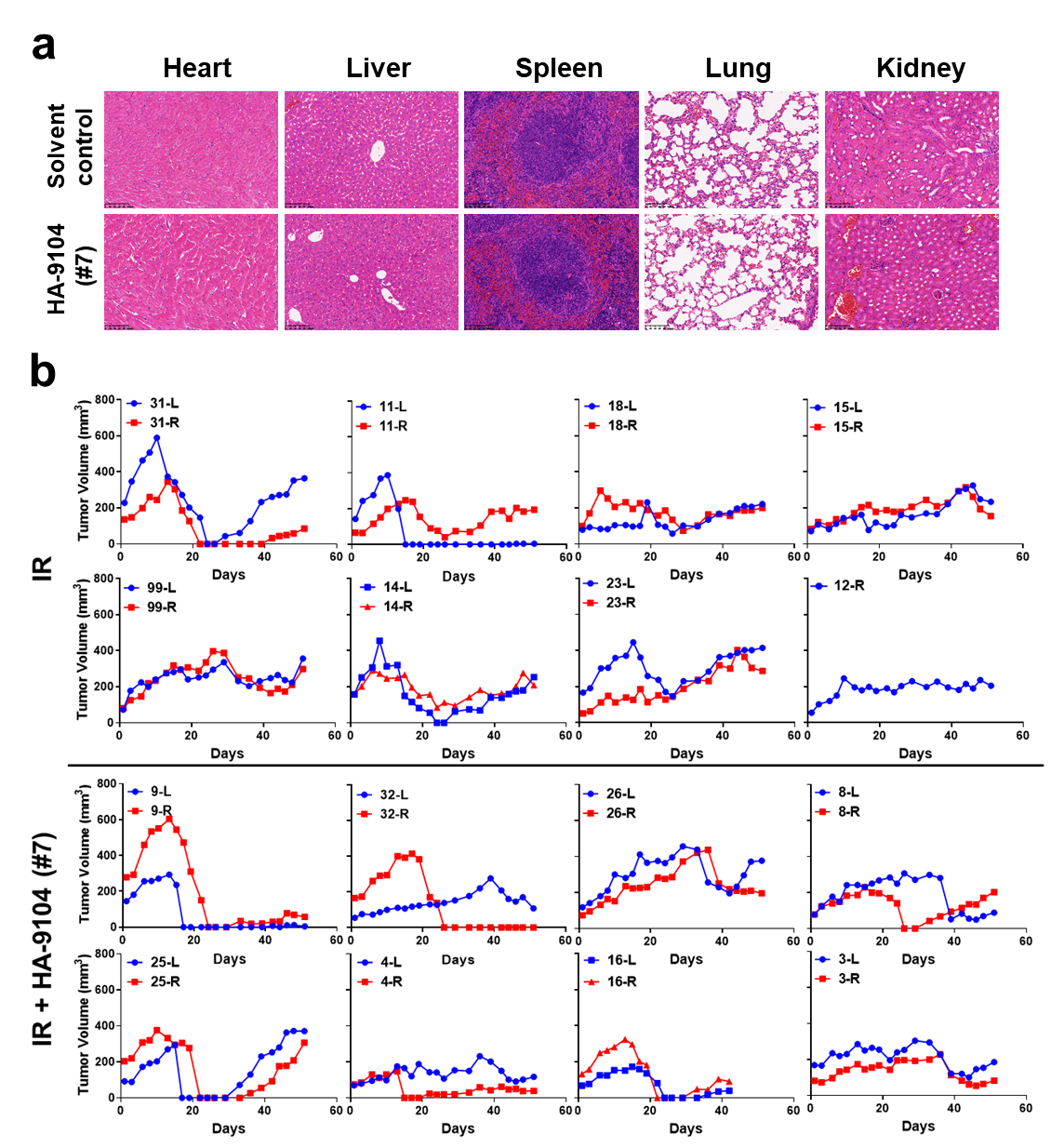
**

**Figure. S6 HA-9104 suppresses tumor growth and enhances radiosensitivity *in vivo.***

**a** H&E staining of indicated organs from the mice treated with solvent control or HA-9104 (**#7**). **b** The growth curves of tumors from the individual mice in *in vivo* radiosensitization assay. IR, radiation.
